# Construction of an expression platform for fungal secondary metabolite biosynthesis in *Penicillium crustosum*

**DOI:** 10.1007/s00253-024-13259-3

**Published:** 2024-07-24

**Authors:** Jenny Zhou, Xiaoling Chen, Shu-Ming Li

**Affiliations:** Institut Für Pharmazeutische Biologie Und Biotechnologie, Fachbereich Pharmazie, Philipps-Universität Marburg, Robert-Koch-Straße 4, 35037 Marburg, Germany

**Keywords:** *Penicillium crustosum*, Fungal host, Heterologous expression, Genetic dereplication, Secondary metabolite biosynthesis, Natural products

## Abstract

**Abstract:**

Filamentous fungi are prolific producers of bioactive natural products and play a vital role in drug discovery. Yet, their potential cannot be fully exploited since many biosynthetic genes are silent or cryptic under laboratory culture conditions. Several strategies have been applied to activate these genes, with heterologous expression as one of the most promising approaches. However, successful expression and identification of new products are often hindered by host-dependent factors, such as low gene targeting efficiencies, a high metabolite background, or a lack of selection markers. To overcome these challenges, we have constructed a *Penicillium crustosum* expression host in a *pyrG* deficient strain by combining the split-marker strategy and CRISPR-Cas9 technology. Deletion of *ligD* and *pcribo* improved gene targeting efficiencies and enabled the use of an additional selection marker in *P. crustosum*. Furthermore, we reduced the secondary metabolite background by inactivation of two highly expressed gene clusters and abolished the formation of the reactive *ortho*-quinone methide. Finally, we replaced the *P. crustosum* pigment gene *pcr4401* with the commonly used *Aspergillus nidulans wA* expression site for convenient use of constructs originally designed for *A. nidulans* in our *P. crustosum* host strain. As proof of concept, we successfully expressed a single polyketide synthase gene and an entire gene cluster at the *P. crustosum wA* locus. Resulting transformants were easily detected by their albino phenotype. With this study, we provide a highly efficient platform for heterologous expression of fungal genes.

**Key points:**

- *Construction of a highly efficient Penicillium crustosum heterologous expression host*
- *Reduction of secondary metabolite background by genetic dereplication strategy*
- *Integration of wA site to provide an alternative host besides Aspergillus nidulans*

**Graphical Abstract:**

**Figure:**
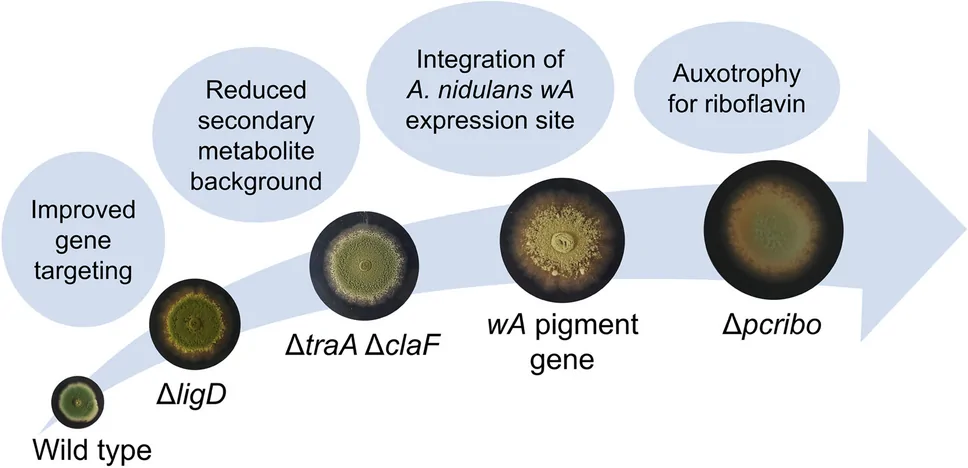

**Supplementary Information:**

The online version contains supplementary material available at 10.1007/s00253-024-13259-3.

## Introduction

Natural products, due to their chemical diversity and broad spectrum of pharmacological effects, have been proven to be one of the most promising sources for the development of new drugs (Atanasov et al. 2021). A well-known example is the discovery of the antibiotic penicillin from *Penicillium rubens* in 1929, which represented a major breakthrough in drug research and enabled the treatment of life-threatening infections (Abdel-Razek et al. 2020). Other examples for bioactive compounds derived from fungi are the antifungal drug griseofulvin, produced by *Penicillium griseofulvum*, the immunosuppressant mycophenolic acid, isolated from *Penicillium brevicompactum*, and the cholesterol-lowering agent lovastatin, produced by *Aspergillus terreus* (Oxford et al. 1939; Freedman et al. 2020; Itoh et al. 2018). However, studies show that only < 10% of biosynthetic gene clusters (BGCs) responsible for the formation of natural products are expressed under usual laboratory conditions, demonstrating the great potential of discovering new bioactive compounds by activation of the large number of silent BGCs (Oikawa 2020). Strategies to activate these cryptic gene clusters include overexpression or deletion of pathway-specific regulators, promoter exchange, or the one strain many compounds (OSMAC) approach by systematic variation of the culture conditions (Xu et al. 2022; Cheng et al. 2021). Nevertheless, these strategies are often hindered by a slow growth of the native producer, demanding culture conditions or a lack of genetic tools available for these strains (Alberti et al. 2017).

A common approach to overcome these difficulties is to express a single gene or entire cluster in a host organism. For this purpose, several model fungi have been developed by expanding the genetic manipulation tools and enhancing gene targeting efficiency, e.g. by deletion of *ku70*, *ku80* and *ligD* homologues (Zhou and Li 2021; Bugeja et al. 2012; Xu et al. 2014). As a part of the repair mechanism for double-strand breaks, the Ku70/80 dimer and LigD activate the non-homologous end joining recombination (NHEJ) pathway, which leads to ectopic DNA integration events (Bugeja et al. 2012). Another approach to enable precise genetic manipulation is the CRISPR-Cas9 technology (Pohl et al. 2016). In this process, the Cas9 endonuclease is directed to a specific DNA target by guideRNA (gRNA), consisting of CRISPR-RNA (crRNA) and trans-activating CRISPR-RNA (tracrRNA). The crRNA contains a 20-nucleotide spacer sequence, which defines the DNA target, flanked by a short protospacer adjacent motif (PAM), and a scaffold region. In contrast, the tracrRNA is required for the recognition and binding of Cas9 to the crRNA (Woodcraft et al. 2023). Provided with gRNA, Cas9 can recognise genomic sequences complementary to the spacer and introduce site-specific cuts in the target DNA (Tong et al. 2019). The resulting double-strand break facilitates targeted genome editing events, such as insertion, deletion, or mutation of genes. CRISPR-Cas9-based gene manipulation has recently been implemented for several fungal species including *Aspergillus nidulans* (Caesar et al. 2020), *Penicillium polonicum* (Valente et al. 2021), *Penicillium verruculosum* (Kislitsin et al. 2023), *Penicillium rubens* (Gil-Durán et al. 2023) and *Penicillium chrysogenum* (Pohl et al. 2016).

Despite being easily genetically manipulated, identification of novel secondary metabolites (SMs) of cryptic or silent genes after heterologous expression in a host organism can be challenging if the host itself owns a large number of active biosynthetic genes. First, interpretation of gene or gene cluster function becomes more difficult as the products can be further modified by host enzymes or by coupling with reactive intermediates (Xiang et al. 2020; Liao et al. 2020). In addition, compounds that only appear as minor products in the cultures of the heterologous expression transformants can be hardly detected if the host has a high SM background. Furthermore, high expression of endogenous biosynthetic pathways in the host organism can result in competition for substrates like acetyl-CoA and malonyl-CoA, therefore limiting the activity of the introduced pathways (Itoh et al. 2018; Chen et al. 2020). To overcome these challenges and to eliminate host-dependent product formation, suitable expression hosts were developed by so-called ‘genetic dereplication’ strategies, in which major known SM biosynthetic gene clusters were inactivated by deletion of genes or whole BGCs (Chiang et al. 2016). The most prominent example is the *A. nidulans* strain LO8030, in which deletion of eight highly expressed SM gene clusters significantly reduced the SM background (Chiang et al. 2016). Other examples include *Aspergillus oryzae* and the fungicolous fungus *Calcarisporium arbuscula* (Dao et al. 2021; Cheng et al. 2021).

*Penicillium crustosum* is a fast-growing, ubiquitously occurring filamentous fungus. It can grow on a variety of foods, including fruits, corn, meat and cheese, and is therefore regarded as a major contributor to food spoilage (Pitt 2002). To date, a large number of SMs have been isolated from different *P. crustosum* strains, including alkaloids, polyenes and diketopiperazines (Liu et al. 2018; Su et al. 2014). From a biotechnological point of view, *P. crustosum* strains were proven to be prolific producers of lipases, tannases, cellulases and xylanases, which are widely used as biocatalysts for various industrial applications (Espinoza-Abundis et al. 2023; Bittencourt et al. 2020; Batra and Saxena 2005; Hasnaoui et al. 2022). The* P. crustosum* strain PRB-2 used in this study was first isolated from deep-sea sludge in Prydz Bay (Wu et al. 2012). The predominant products identified in PRB-2 were reported to be terrestric acid, clavatol, hydroxyclavatol and their derivatives peniphenones and penilactones (Fan et al. 2019).

One of the major advantages of using *P. crustosum* as a heterologous host is the rapid and efficient sporulation, which facilitates genetic manipulation and downstream applications. A comparison of eight different *Aspergillus* and *Penicillium* species revealed that *P. crustosum* was the fastest to sporulate and yielded the highest number of spores per colony (Leggieri et al. 2018). Another study focused on the adaptation of *P. crustosum* to various stress factors (Kalinina et al. 2017). The results demonstrated that *P. crustosum* was able to retain growth and SM production throughout numerous changes in environmental and nutritional factors, such as media composition, temperature, pH and light exposure. Given that heterologous hosts are required to adapt to different laboratory growth conditions, this work further emphasises the suitability of constructing *P. crustosum* as an expression platform for SM biosynthesis. Finally, the functionality of *P. crustosum* PRB-2 as heterologous host was confirmed by successful expression of genes from *Penicillium camemberti*, *Penicillium brevicompactum* and *Aspergillus ruber* (Kindinger et al. 2019).

In a previous study, the recyclable marker *pyrG* has already been deleted in PRB-2 to facilitate genetic manipulation. Furthermore, the gene locus *pcr4401*, coding for a polyketide synthase (PKS) involved in spore pigment formation, was demonstrated to be a suitable integration site for heterologous gene expression (Kindinger et al. 2019).

In the present study, we have optimised *P. crustosum* PRB-2 in various aspects, including gene targeting efficiency, reduction of secondary metabolite background and choice of selectable markers, to offer a valuable alternative to the existing heterologous expression systems. For this purpose, we have combined the split-marker-based *pyrG* recycling strategy (Zhou and Li 2021) with CRISPR-Cas9 to enable multiple rounds of highly efficient genetic manipulation followed by convenient selection marker recycling. With this approach, we have successfully deleted numerous genes, including *ligD*, the riboflavin synthase gene *pcribo* and backbone genes of the two dominant biosynthetic pathways in PRB-2. In addition, we replaced the spore pigment gene *pcr4401* with the *wA* integration site from the commonly used *A. nidulans* LO8030 host. This provides an alternative platform for the expression of constructs that do not lead to sufficient product formation in LO8030. To the best of our knowledge, there is no report on the replacement of a pigment-based integration site to allow the use of the same constructs in different fungal hosts prior to this study. As proof of concept, constructs designed for LO8030 were successfully used to express a PKS gene and an entire BGC in the constructed strain. With this study, we provide a new *P. crustosum* platform for heterologous expression of fungal genes, especially those for SM production.

## Materials and methods

### Strains, media and culture conditions

*P. crustosum* strains used and generated in this work are listed in Table 1. *A. nidulans* strains used in this work are listed in Supplemental Table S1.

**Table 1:** *Penicillium crustosum* strains used and generated in this study

Strain	Genotype
PRB-2	Wild type (Wu et al. 2012)
FK15	Δ*pyrG* in PRB-2 (Kindinger et al. 2019)
JZ03	Δ*ligD* (*pcr2372*)::*pyrG* in FK15
JZ04	Δ*pyrG* in JZ03
JZ05	Δ*traA* (*pcr11009*)::*pyrG* in JZ04
JZ06	Δ*pyrG* in JZ05
JZ32	Δ*claF* (*pcr3094*)::*pyrG* in JZ06
JZ35	Δ*pyrG* in JZ32
JZ37	Δ*pcr4401*::*wA*:*pyrG* in JZ35
JZ38	Δ*pyrG* in JZ37
JZ39	Δ*wA*::*gpdA(p)*:*pyrG* in JZ38
JZ40	Δ*wA*::*gpdA(p)*:*orsA*:*pyrG* in JZ38
JZ51	Δ*pcribo* (*pcr11223*)::*pyrG* in JZ38
JZ52	Δ*pyrG* in JZ51
JZ54	Δ*wA*::*gpdA(p)*:*anuA–anuK*:*afriboB* in JZ52
JZ56	Δ*wA*::*gpdA(p)*:*afriboB* in JZ52

*Saccharomyces cerevisiae* and *Escherichia coli* strains used for plasmid cloning and DNA propagation were grown and selected as reported previously (Nies and Li 2021). Fungal strains were cultivated on GMM agar plates (1.0% glucose, 50 mL/L salt solution, 1 mL/L trace element solution and 1.6% agar) at 25 °C (*P. crustosum*) or 37 °C (*A. nidulans*) for sporulation and in PDB medium (24 g/L potato dextrose broth) or rice medium (20 g royal tiger rice, 30 ml water) at 25 °C for secondary metabolite production. Appropriate supplementation with 0.5 g/L uridine, 0.5 g/L uracil, 5 mg/L riboflavin and/or 0.5 mg/L pyridoxine was added as required.

*P. crustosum* strains PRB-2 and JZ52 were deposited as strains MMBC-MIR-00001206 and MMBC-MIR-00008263, respectively, in the culture collection of the Marine Medicinal Bioresource Center (MMBC), Ocean University of China, Qingdao, the People’s Republic of China. PRB-2 is also available as strain MFCSCS 200 in the culture collection of the Key Laboratory of Marine Drugs, Ocean University of China, Qingdao, the People’s Republic of China (Zhou and Li 2021).

### Genome sequencing and sequence analysis

Genome sequencing and sequence analysis were carried out as described previously (Fan et al. 2019). The Whole Genome Shotgun project of *P. crustosum* PRB-2 including the reported sequences has been deposited at DDBJ/ENA/GenBank under the accession JBCAMJ000000000. The version described in this paper is version JBCAMJ010000000. The sequence of the *A. nidulans wA* gene was reported previously (Watanabe et al. 1999).

### Isolation of genomic DNA, PCR amplification and cloning

Plasmids and primers used and generated in this work are listed in Supplemental Tables S2 and S3.

Isolation of genomic DNA, PCR amplification and cloning were performed according to protocols described previously (Fan et al. 2019).

### Construction of deletion plasmids

For gene deletion in this study, two split-marker plasmids were constructed according to previous protocols (Zhou and Li 2021). The first plasmid contains the approximately 1.5 kb upstream flanking sequence of the target gene and two-thirds of the *pyrG* marker (1145 bp) from the 5′-end. In addition, an approximately 0.3 kb fragment of the downstream flanking sequence was cloned between the upstream sequence and the *pyrG* marker to enable subsequent marker recycling based on homologous recombination. The second split-marker plasmid consists of two-thirds (1140 bp) of the *pyrG* marker from the 3′-end and the approximately 1.5 kb downstream DNA sequence of the target gene. All fragments were amplified via PCR and assembled via homologous recombination in *S. cerevisiae*.

### Construction of plasmids for replacement of *pcr4401* by the *wA* gene and its flanking regions

For heterologous expression of *wA* and its flanking regions at the *P. crustosum pcr4401* locus with subsequent *pyrG* marker recycling, the expression cassette was constructed using two split-marker plasmids. The first consists of approximately 1.5 kb of the *pcr4401* upstream sequence, 1 kb of the *wA* upstream sequence, one part of the *wA* gene (3581 bp) and two-thirds of the *pyrG* sequence at the 5′-end (1145 bp) with overlapping regions to the *Sma*I cloning site of the pESC-URA vector. The second split-marker plasmid carries two-thirds of the *pyrG* gene at the 3′-end (1140 bp), the other part of the *wA* gene (3412 bp including approximately 300 bp overlap to the first half) with its 1 kb downstream sequence and approximately 1.5 kb of the *pcr4401* downstream sequence, and cloned into the *Sma*I site of pESC-URA via homologous recombination.

### Construction of the *P. crustosum* 5S rRNA promoter-driven gRNA expression vector

For construction of the vector to express Cas9 and gRNA, the 5S rRNA promoter sequence was amplified from genomic DNA of *P. crustosum* PRB-2 and inserted into the *Kpn*I and *Bam*HI restriction sites of the pCas9-tRp-gRNA (Liang et al. 2018) vector via homologous recombination.

### Selection of protospacers and construction of the gRNA expression cassettes

gRNA spacer sequences are listed in Supplemental Table S4. gRNA spacer design and off-target predictions were performed using CHOPCHOP (Labun et al. 2019) and EuPaGDT (Peng and Tarleton 2015). Off-target searches were conducted using the Local BLAST tool from BioEdit Sequence Alignment Editor (Hall 1999) and the genomic sequence of *P. crustosum* PRB-2 as reference. gRNA spacers were designed as 27-bps sense and antisense oligonucleotides including the 20-bps spacer sequence, the 3-bps PAM sequence and 4-bps overlapping sequence to the *Bsm*BI restriction site of the pJZ65 vector (Arévalo et al. 2024; Zheng et al. 2014; Molina-Risco et al. 2021) and synthesised by Seqlab GmbH (Göttingen, Germany). Then, 1 µL of both sense and antisense nucleotides (10 µM) was annealed after adding 48 µL annealing buffer in a thermocycler, first at 95 °C for 5 min, and then reduced to 25 °C at a rate of 1 °C/min according to protocols described previously (Zheng et al. 2014). The annealed gRNA spacers were then cloned into the *Bsm*BI-linearised vector pJZ65 via ligation using the T4 DNA Ligase system from Promega GmbH (Walldorf, Germany) and amplified in *E. coli*. A schematic illustration of a Cas9 and gRNA expression plasmid used in this work is given in Supplemental Fig. S1.

### Genetic manipulation in *P. crustosum* strains

Transformation of *P. crustosum* strains was performed by polyethylene glycol (PEG)-mediated protoplast transformation as described previously (Zhou and Li 2021; Janzen et al. 2023). Protoplast formation for the deletion of *ligD* and *traA* was conducted using an enzyme mixture consisting of 10 mL osmotic buffer (1.2 M MgSO4 in 10 mM sodium phosphate, pH 5.8), 50 mg lysing enzyme from *Trichoderma harzianum* (Sigma-Aldrich, St. Louis, USA) and 20 mg yatalase from *Corynebacterium* sp. OZ-21 (OZEKI Co., Ltd., Hyogo, Japan). For the deletion of *claF* and *pcribo* and the expression of *wA*, *orsA* and *anuA–K*, due to the unavailability of the aforementioned lysing enzymes, we used an enzyme mixture consisting of 100 μL cellulase TXL, 20 μL chitinase, 5 μL protease (all from ASA Spezial Enzyme GmbH, Wolfenbüttel, Germany), 25 mg lysing enzyme from *Aspergillus* sp. (Sigma-Aldrich, St. Louis, USA) and 20 mg yatalase in 10 mL osmotic medium. Germlings were incubated in the enzyme mixtures at 30 °C, 100 rpm for 2.5 h for cell wall degradation. Protoplasts were collected by overlaying with trapping buffer (0.6 M sorbitol in 0.1 M Tris-HCI, pH 7.0) and centrifuging at 5000 rpm for 15 min. Finally, the protoplasts were resuspended in STC buffer (1.2 M sorbitol, 10 mM CaCl_2_ and 10 mM Tris-HCI, pH 7.5) to a final concentration of approximately 1 × 10^8^ protoplasts/mL and transformed according to previous protocols (Zhou and Li 2021).

For CRISPR-Cas9 mediated gene editing, the transformation system contained 100 µL protoplast suspension, 5 µg of the Cas9-gRNA expression construct and 4 µg of each split-marker deletion/expression cassette (amplified via PCR and purified using ethanol precipitation) and was treated following a previous protocol (Gao et al. 2017) with adjustments for *P. crustosum*. Solution S3 was modified by using 60% PEG 4000 and 50 mM Tris–HCl. In addition, the transformation system was mixed with 8 mL SMM top medium at 55 °C before being poured onto plates containing 15 mL SMM bottom medium, both media supplemented as required for selection (Zhou and Li 2021). After cultivation at 25 °C for 5–7 days, transformants were obtained.

### Verification of transformants, cultivation and LC–MS analysis of secondary metabolites

PCR verification of transformants, cultivation for secondary metabolite production and LC–MS analysis of secondary metabolites were conducted as described previously (Zhou and Li 2021). Cultures in PDB and/or rice medium were extracted with ethyl acetate after 7, 10 and 14 days for detection of secondary metabolites. All cultivation experiments for the quantification of compound production were performed with three individual transformants.

## Results

### Identification of *ligD* homologue in *P. crustosum* and construction of Δ*ligD* mutant for improved gene targeting

**Fig. 1:**
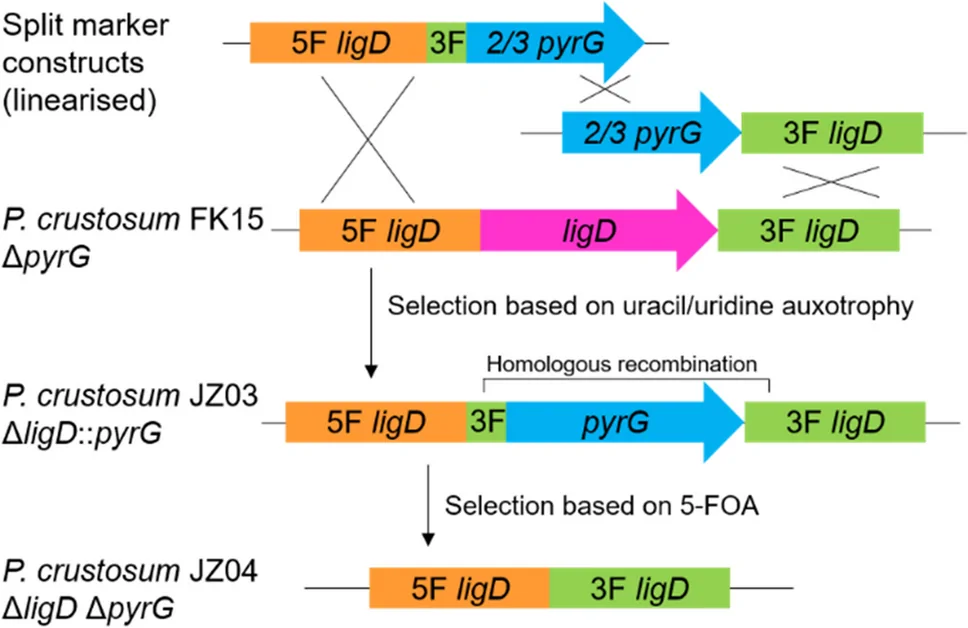
Split-marker strategy for the deletion of *ligD* with subsequent selection marker recycling (5F: upstream flanking region, 3F: downstream flanking region)

First, we aimed to enhance the gene targeting efficiency in *P. crustosum* by inactivation of the NHEJ pathway via deletion of the gene coding for LigD (DNA ligase IV). In fungi, the NHEJ repair mechanism is initiated by a complex network of enzymes, including the DNA ligase IV and a DNA-dependent protein kinase, which includes the Ku70/80 subunits. In a previous study, a Δ*ku70* mutant was constructed with the objective of enhancing the gene targeting efficiencies in *P. crustosum* (Zhou and Li 2021). However, several studies have indicated the presence of genomic defects and reduced growth rates in other Δ*ku70 Penicillium* strains (Bugeja et al. 2012; Snoek et al. 2009). Given that these defects were not reported for Δ*ligD* strains, we proceeded to construct a *P. crustosum* expression host with a Δ*ligD* background. To identify a LigD homologue in *P. crustosum*, PoligD (ARQ16397) (Qin et al. 2017) from *Penicillium oxalicum* was used as a query for BLAST sequence alignment (http://blast.ncbi.nlm.nih.gov). This led to the identification of Pcr2372 from *P. crustosum* PRB-2, consisting of 999 amino acid residues, with a sequence identity of 73.3%. BLAST analysis showed that Pcr2372 shares 91.2%, 77.6% and 77.5% identities with the putative Lig4 homologues from *Penicillium digitatum*, *Penicillium canariense* and *Penicillium brasilianum*, respectively (Supplemental Figs. S2–S4). Pcr2372 was therefore designated as LigD. The constructs for the deletion of *ligD* were designed using the split-marker strategy as described previously with *pyrG* as selection marker (Fig. 1) (Zhou and Li 2021). Based on bidirectional selection, *pyrG* can be easily recycled and used for multiple rounds of gene manipulation experiments. Transformation of *P. crustosum* Δ*pyrG* strain FK15 (Kindinger et al. 2019) with the linearised constructs pJZ03 and pJZ05 for the deletion of *ligD* resulted in Δ*ligD*::*pyrG* strain JZ03 (Table 1). Transformants were selected on medium without uracil and uridine and identified via PCR using primers either binding outside of the deletion construct or in the *pyrG* marker gene, resulting in approximately 2.5 kb fragments for the deletion mutants and no PCR product in the wild-type strain (Supplemental Fig. S5). Subsequently, selection of JZ03 on medium containing 5-fluoroorotic acid (5-FOA), uracil and uridine led to deletion of the *pyrG* marker based on recombination of the short homologous sequences flanking the marker gene (Fig. 1), resulting in 26 colonies for the Δ*ligD*Δ*pyrG* strain JZ04. Eight mutants were further subjected to verification via PCR. All transformants were confirmed by amplification of the entire gene locus either with or without the *pyrG* gene and by proving the absence of *pyrG* (Supplemental Fig. S5). As proof of concept, *P. crustosum* FK15 (Δ*pyrG*), JZ02 (Δ*ku70*Δ*pyrG*)(Zhou and Li 2021) and JZ04 (Δ*ligD*Δ*pyrG*) were transformed with pFK24. In a previous study, pFK24 was used for the expression of a PKS gene at the *pcr4401* locus, which allows for transformant detection based on their phenotype (Kindinger et al. 2019). Gene targeting efficiencies for three independent transformations were calculated to be 12.3%, 36.4% and 42.0% for FK15, JZ02 and JZ04, respectively.

### Generation of Δ*traA*::*pyrG* and Δ*traA*Δ*pyrG* mutant strains JZ05 and JZ06 for reduction of the secondary metabolite background

**Fig. 2:**
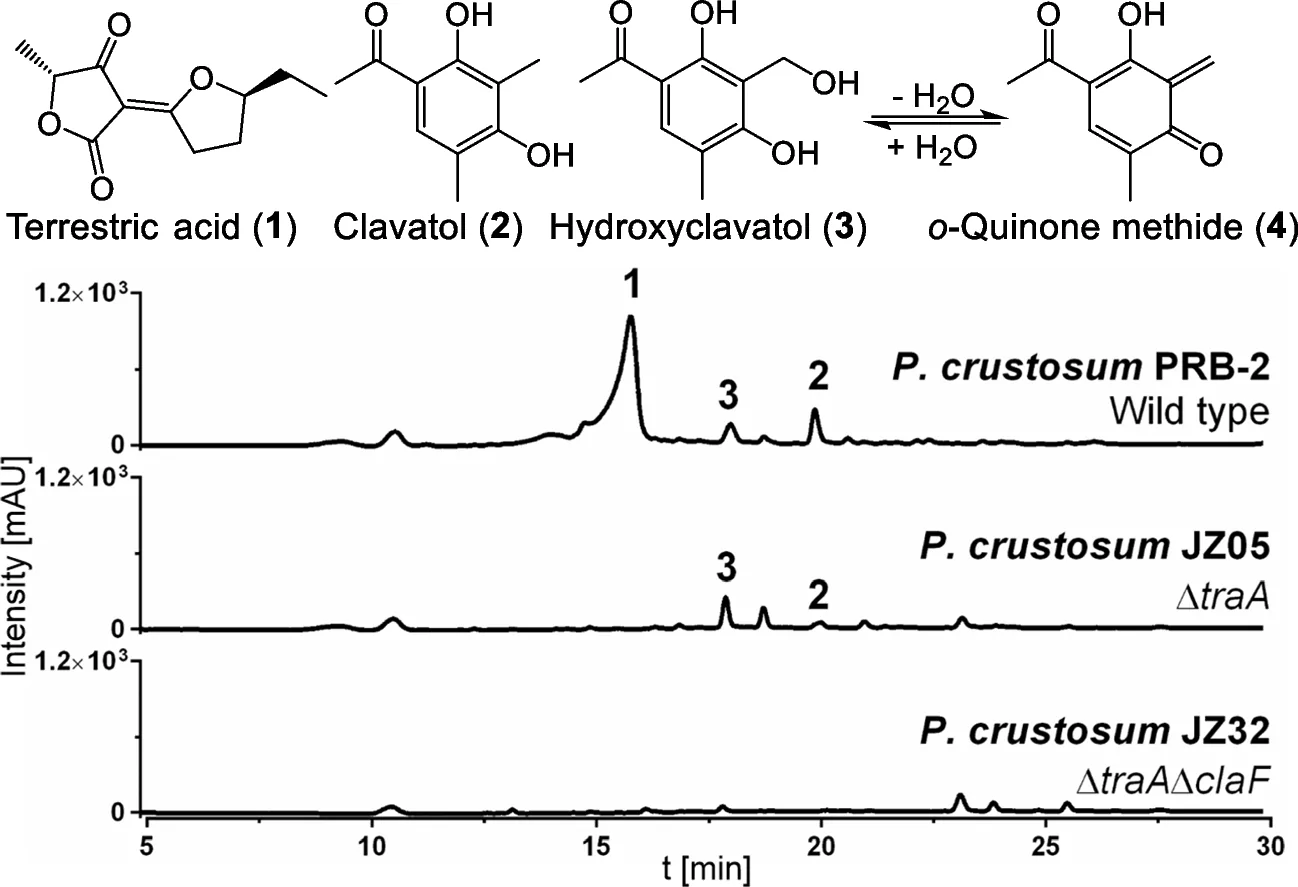
LC–MS results of EtOAc extracts from *P. crustosum* PRB-2 (wild type), JZ05 (Δ*ligD*Δ*traA*) and JZ32 (Δ*ligD*Δ*traA*Δ*claF*) after 14 days of cultivation in PDB medium. Absorptions at UV 254 nm are displayed. [M + H]^+^ ions of **1** and **2** were detected at m/z 211.1013 and 181.0860, respectively. The [M + Na]^+^ ion for **3** was detected at m/z 219.0644

Previous studies have shown that *P. crustosum* predominantly produces terrestric acid (**1**) (Fig. 2) (Fan et al. 2019). Hence, for generation of a strain with a reduced SM background, the gene *traA* (*pcr11009*) was chosen for deletion. *traA* codes for a PKS-NRPS hybrid enzyme, which has previously been proven to catalyse the formation of (5*S*)-carboxylcrustic acid, which is subsequently converted to **1** by tailoring reactions (Fan et al. 2020). As a result, deletion of *traA* should abolish the production of **1**. The deletion plasmids pJZ06 and pJZ07 were prepared according to the strategy described previously (Zhou and Li 2021). Transformation of *P. crustosum* JZ04 with the linearised deletion constructs generated Δ*traA*::*pyrG* strain JZ05 after selection based on uracil/uridine auxotrophy. Subsequently, *pyrG* marker recycling was achieved by selection on 5-FOA containing medium as described above, yielding Δ*traA*Δ*pyrG* strain JZ06. Transformants were confirmed via PCR (Supplemental Fig. S6). To verify the loss of terrestric acid production, JZ05 was cultivated in PDB medium for 14 days and SMs were extracted with ethyl acetate. LC–MS analysis of the extracts revealed that the production of terrestric acid was abolished in JZ05 (Fig. 2).

### Generation of Δ*claF*::*pyrG* and Δ*claF*Δ*pyrG* mutant strains JZ32 and JZ35 for prevention of *ortho*-quinone formation

As expected, abolishment of **1** led to the detection of clavatol (**2**) and hydroxyclavatol (**3**) in Δ*traA* strain JZ05 as major products, as reported previously (Fig. 2) (Fan et al. 2019). Moreover, **3** was proven to spontaneously convert to the *ortho*-quinone methide (**4**), which acts as a highly reactive coupling reagent for a broad variety of natural products (Liao et al. 2020; Wu et al. 2022; Fan et al. 2019; Doyon et al. 2019). Production of **3** and **4** could therefore hinder the identification of novel SMs after gene expression in *P. crustosum*. Hence, *claF* (*pcr3094*), coding for a nonreducing PKS in the biosynthesis of **2**–**4** was chosen for gene deletion. The deletion plasmids pJZ23 and pJZ34 were constructed according to the aforementioned split-marker strategy (Zhou and Li 2021) and used for the deletion of *claF* in the Δ*traA*Δ*pyrG* strain JZ06. However, changes in the enzyme mixture used for PEG-mediated protoplast transformation led to a decrease in the quality and quantity of fungal protoplasts. Consequently, CRISPR-Cas9-assisted transformation was tested to enhance transformation efficiencies.

Since the CRISPR-Cas9-mediated gene editing tool has not yet been implemented for *P. crustosum*, a suitable promoter for the expression of gRNA needed to be identified first. As reported for *P. oxalicum* and *Trichoderma reesei*, the 5S rRNA promoter enabled gRNA expression in both strains (Wang et al. 2021). In addition, it was reported that for *T.* *reesei*, employment of the endogenous 5S rRNA promoter instead of heterologous promoters proved to be more efficient for gene editing. To identify the *P.* *crustosum* 5S rRNA promoter, the known 5S rRNA sequences deposited in the 5SRNAdb (Szymanski et al. 2016) for *P.* *chrysogenum* and *P.* *griseofulvum* were used as queries to search for a homologue in the *P.* *crustosum* genome using online BLAST approaches. Sequence comparison revealed that the genome of *P. crustosum* contains a 5S rRNA promoter sequence with 100% identity on the nucleic acid level to the sequences of the related *Penicillium* strains mentioned above (Supplemental Fig. S7). Therefore, the 5S rRNA promoter was amplified from *P.* *crustosum* PRB-2 and inserted into the pCas9-tRp-gRNA (Liang et al. 2018) vector, resulting in the gRNA expression vector pJZ65. The gRNA spacer for targeting *claF* was predicted using CHOPCHOP (Labun et al. 2019) and EuPaGDT (Peng and Tarleton 2015) and cloned into the linearised pJZ65 vector, yielding pJZ66 (Supplemental Fig. S1). CRISPR-Cas9-assisted transformation of *P. crustosum* JZ06 with pJZ66 and the PCR-amplified deletion cassettes from pJZ23 and pJZ34 finally resulted in Δ*claF*::*pyrG* strain JZ32. Cultivation in PDB medium for 14 days, followed by extraction with ethyl acetate and LC–MS analysis, revealed the abolishment of **2** and **3** (Fig. 2). Subsequently, the *pyrG* selection marker was removed in JZ32 as described above to yield Δ*claF*Δ*pyrG* strain JZ35. All transformants were verified via PCR (Supplemental Fig. S8).

### Replacement of the *P. crustosumpcr4401* pigment gene by the *A. nidulanswA* expression site

**Fig. 3:**
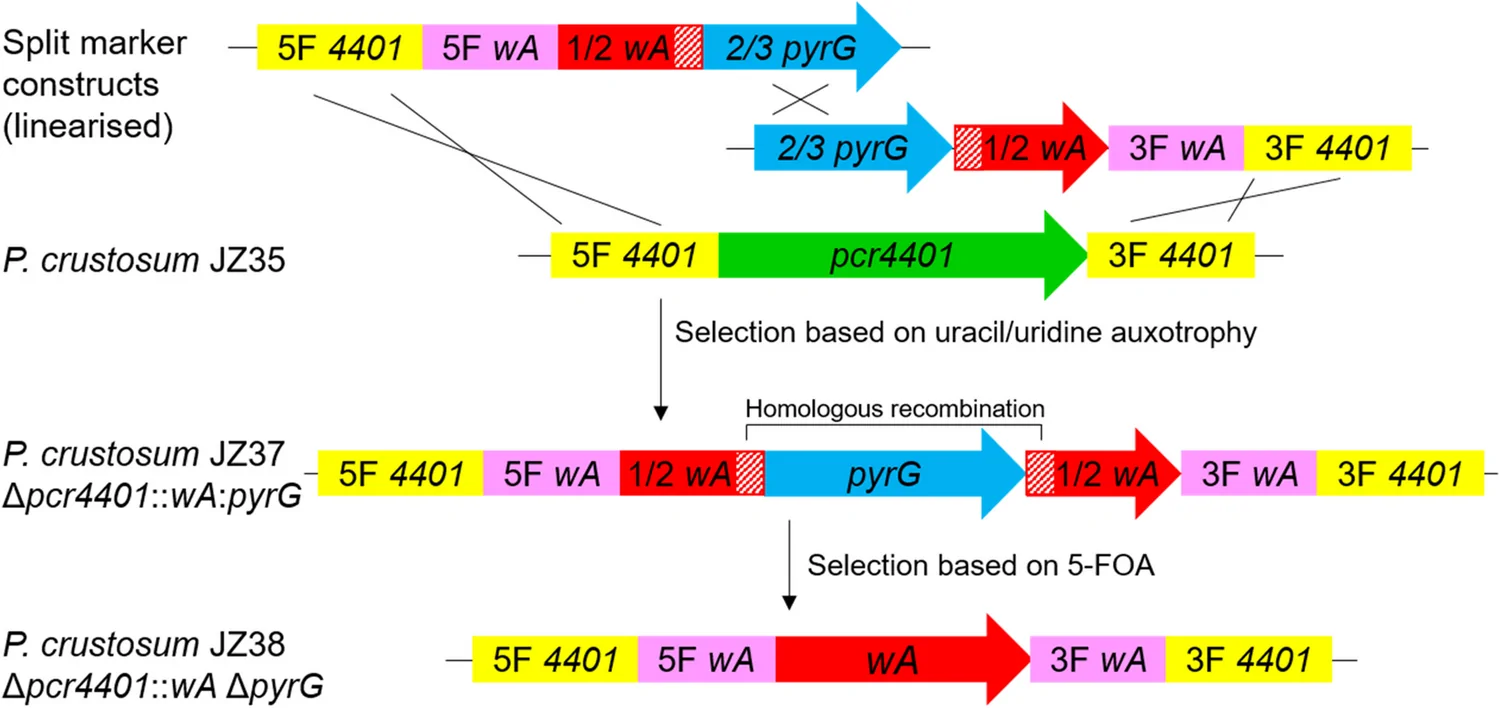
Split-marker strategy for integration of the *wA* gene including its flanking regions in the *P. crustosum pcr4401* locus with subsequent selection marker recycling. The red hatched area indicates homologous regions within the *wA* gene

The objective of this study is the development of a valuable alternative to the currently available heterologous expression systems. One of the most frequently used expression hosts is the genetic dereplication strain *A. nidulans* LO8030 (Chiang et al. 2016). In particular, the *A. nidulans wA* pigment locus represents a common integration site for heterologous expression, as it allows the selection of transformants based on their albino phenotype. To establish *P. crustosum* as an alternative expression host besides *A. nidulans* LO8030, the *A. nidulans wA* integration site was chosen for replacement of the *P. crustosum* pigment gene *pcr4401*. A previous study demonstrated that the protein Pcr4401, sharing 67% sequence identity with the gene product of *wA*, was responsible for the formation of the melanin precursor YWA1 (Kindinger et al. 2019). By replacing *pcr4401* with the *wA* pigment gene and its flanking regions, constructs designed for heterologous expression at the *A.* *nidulans wA* locus can also be used for *P. crustosum*. For this purpose, the *wA* gene including its 1 kb flanking regions was amplified by PCR from genomic DNA of *A.* *nidulans* LO8030 in two parts and cloned into the two split-marker expression plasmids pJZ38 and pJZ39, including two-thirds of *pyrG* and the 0.3 kb repetitive sequence for subsequent selection marker recycling (Fig. 3). In addition, the Cas9 and gRNA expression plasmid pJZ85 was constructed as mentioned before, with the gRNA spacer designed to target the *pcr4401* gene.

**Fig. 4:**
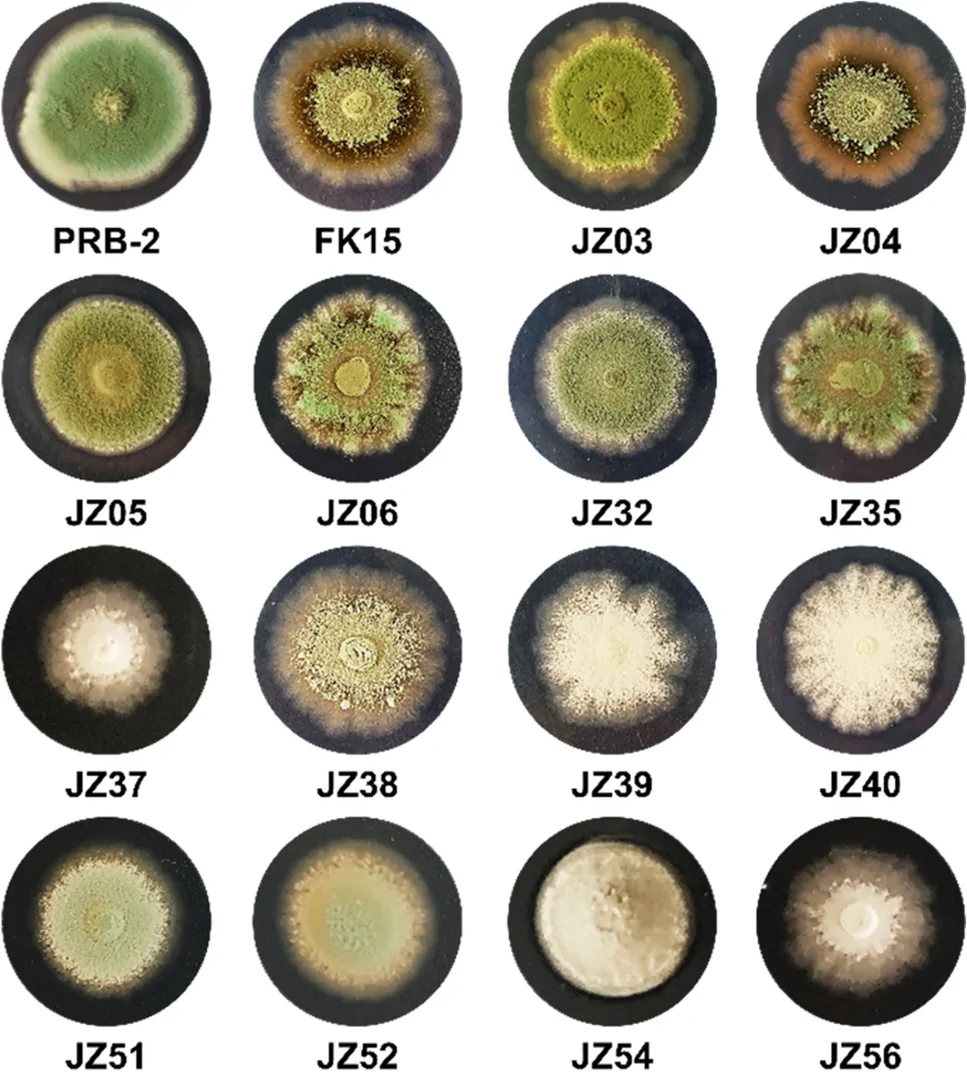
Phenotypes of *P. crustosum* strains grown on GMM with appropriate supplementation for 7 days at 25 °C. Replacement of *pcr4401* and interruption of the *wA* pigment gene by *pyrG* led to the white phenotype in JZ37. Subsequent *pyrG* marker recycling restored the *wA* gene and resulted in pale green conidia in JZ38. Heterologous gene expression in the *wA* locus led to an albino phenotype as observed for strains JZ39, JZ40, JZ54 and JZ56

Transformation of *P. crustosum* JZ35 with pJZ85 and the PCR-amplified expression cassettes from pJZ38 and pJZ39 resulted in two transformants for the *wA* expression strain JZ37, which exhibited a white phenotype due to interruption of the *wA* gene by the *pyrG* sequence (Figs. 3 and 4).

After counterselection on 5-FOA medium, the intact *wA* gene was restored and yielded 22 colonies with green spore pigmentation. Six transformants were further subjected to genotyping and PCR analysis, confirming the presence of the full *wA* gene without the *pyrG* sequence in *wA*Δ*pyrG* strain JZ38 (Supplemental Fig. S9). Compared to JZ35, which harbours the native *P. crustosum pcr4401* pigment gene and produces dark green conidia, the conidia in JZ38 appear more pale green in colour and resemble those reported for *A. nidulans* strains (Fig. 4) (Kindinger et al. 2019).

### Generation of Δ*pcribo*::*pyrG* and Δ*pcribo*Δ*pyrG* mutant strains JZ51 and JZ52 to enable the use of riboflavin-based selection markers

For expanding the repertoire of selectable markers, we aimed to generate a *P. crustosum* strain with a riboflavin auxotrophic background. This provides the possibility to employ the commonly used *A. fumigatus afriboB* (Zhang et al. 2017) gene as a third selectable marker in addition to *pyrG* and the hygromycin B resistance gene (Kindinger et al. 2019). For this purpose, the *A. nidulans riboB* gene, coding for the GTP cyclohydrolase II in the biosynthesis of riboflavin, was used to identify a homologue in *P. crustosum* (Chiang et al. 2013). BLASTx search with the translated nucleotide sequence of *riboB* (AN0670.2) led to the identification of Pcr11223 from *P. crustosum* PRB-2, consisting of 376 amino acid residues, with a sequence identity of 69.5% (Supplemental Fig. S10). Pcr11223 was therefore designated as Pcribo. Gene deletion with the aforementioned split marker strategy (Zhou and Li 2021), using PCR amplified deletion cassettes from pJZ96 and pJZ97 in combination with the Cas9 and gRNA containing plasmid pJZ98, resulted in 18 colonies. Six of them were further genotyped. Subsequent PCR analysis confirmed the absence of *pcribo* in four of the transformants, thereby verifying the Δ*pcribo*::*pyrG* strain JZ51 (Supplemental Fig. S11). Subsequent marker recycling yielded 34 colonies that were able to grow on media containing 5-FOA. Six transformants were genotyped and, by confirmation of the absence of *pyrG* via PCR, resulted in Δ*pcribo*Δ*pyrG* strain JZ52 (Supplemental Fig. S11). Successful deletion of *pcribo* was additionally proven by monitoring the growth only in the presence of riboflavin.

### Expression of constructs designed for *A. nidulans *at *P. crustosum wA *site using *pyrG* as selection marker

**Fig. 5:**
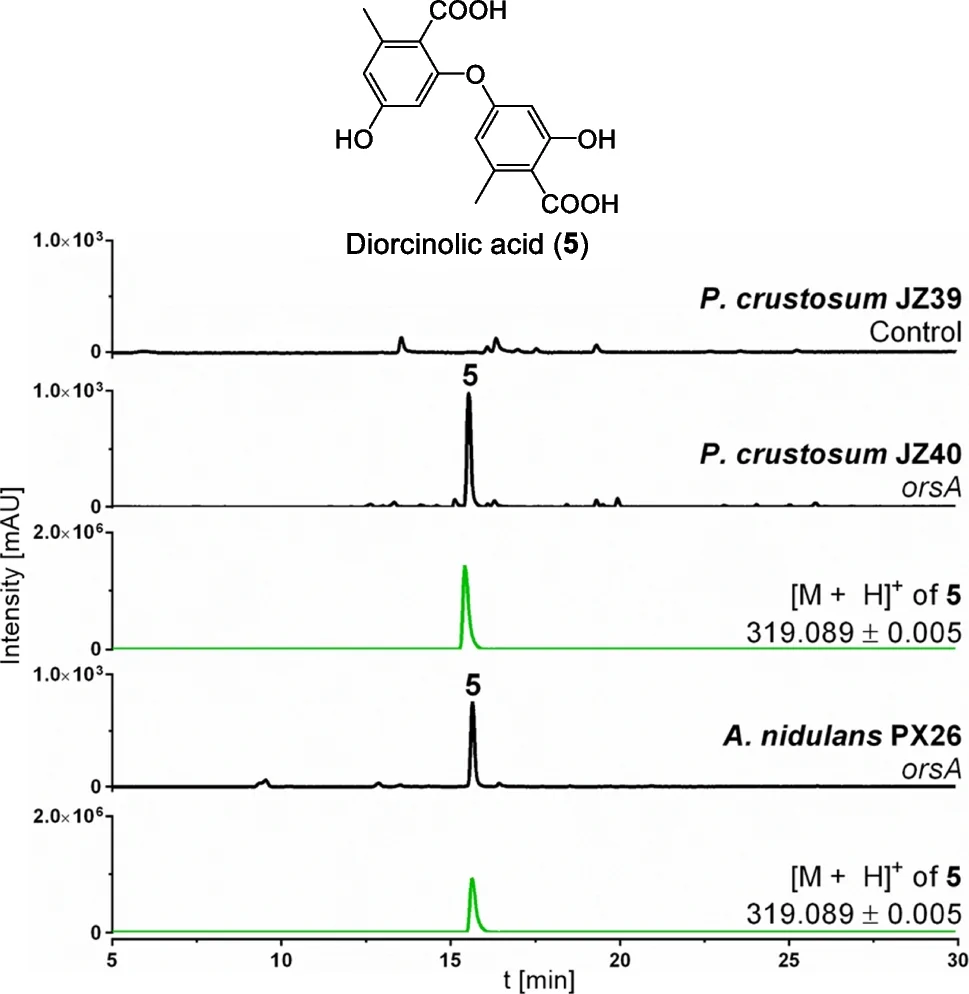
LC–MS results of EtOAc extracts from *P. crustosum* JZ39 (empty vector), JZ40 (*orsA*) and *A. nidulans* PX26 (*orsA*) after 14 days of cultivation in rice medium. Absorptions at UV 254 nm are displayed in black. Extracted ion chromatograms of **5** are illustrated in green

To prove the functionality of gene expression at the *P. crustosum wA* integration site, we selected the plasmid pPX26 (Xiang and Li 2022), which was previously used to express the PKS gene *orsA* in *A. nidulans* LO8030 under the control of the *gpdA* promoter, for expression at the *wA* locus of *P. crustosum* JZ38. After selection, 66 of 90 colonies showed a white phenotype due to replacement of the pigment gene *wA* by the *orsA* expression cassette (Fig. 4). Six of the albino transformants were further confirmed via PCR, resulting in *orsA* expression strain JZ40 (Supplemental Fig. S12). In parallel, expression of the empty vector pYH-wA-pyrG resulted in a total of 61 colonies. Six of the 32 albino mutants were additionally genotyped and confirmed via PCR, yielding control strain JZ39 (Supplemental Fig. S12). After cultivation of three independent transformants on rice medium for 14 days, the *P. crustosum orsA* expression strain JZ40 produced diorcinolic acid (**5**) with a [M + H]^+^ ion at m/z 319.0894, which is consistent with the results of *orsA* expression in *A. nidulans* PX26 (Xiang and Li 2022), whereas **5** could not be detected in empty vector control strain JZ39 (Fig. 5). The yield of **5** produced by JZ40 was estimated to be 1.38 ± 0.06 g per kilogram of rice, which is higher than 0.72 ± 0.01 g per kilogram of rice culture produced by the corresponding *A. nidulans* strain PX26.

### Expression of constructs designed for *A. nidulans* at *P. crustosum wA *site using *afriboB* as a selection marker

**Fig. 6:**
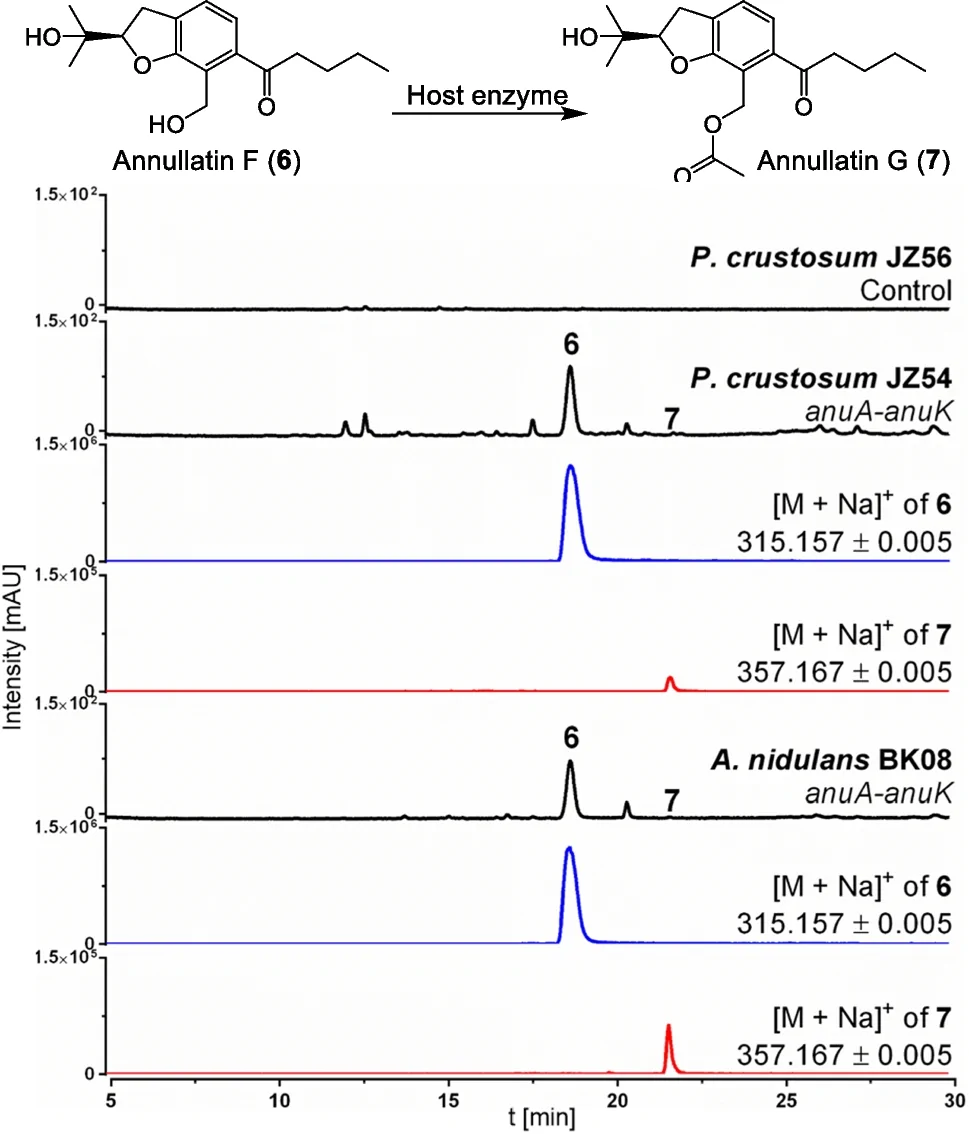
LC–MS results of EtOAc extracts from *P. crustosum* JZ56 (empty vector), JZ54 (*anu* cluster) and *A. nidulans* BK08 (*anu* cluster) after 10 days of cultivation in PDB medium. Absorptions at UV 314 nm are displayed in black. Extracted ion chromatograms of **6** and **7** are illustrated in blue and red, respectively

To prove that the *P. crustosum* host strain is not only able to express individual genes but also entire BGCs, and to confirm that *pcribo* can be replaced with the commonly used *afriboB* selection marker, the *afriboB-*based expression plasmid pBK21, harbouring the genes *anuA*–*anuK* from the known annullatin cluster (*anu*) (Xiang et al. 2022), was chosen for integration at the *P. crustosum wA* site. Transformation of Δ*pcribo* strain JZ52 with the linearised expression construct pBK21 resulted in 79 albino mutants. Six transformants were further subjected to genotyping and PCR analysis, thereby confirming the *anuA*–*K* expression strain JZ54 (Supplemental Fig. S13). As a control, the empty vector pJN017 (Kindinger et al. 2019) was used for transformation to generate control strain JZ56. As expected, transformants showed a white phenotype due to replacement of the *P. crustosum wA* pigment gene (Fig. 4). After confirmation via PCR (Supplemental Fig. S13), three independent transformants of both strains were cultivated in PDB medium for 10 days. LC–MS analysis of the culture extracts revealed the presence of annullatin F (**6**) with a [M + Na]^+^ ion at m/z 315.1565 as the predominant metabolite in JZ54, which was not detected in the isogenic control strain JZ56 (Fig. 6). In parallel, the *A. nidulans anuA*–*K* expression strain BK08 was cultivated and extracted under the same conditions. Comparison of the *P. crustosum* and *A. nidulans* expression strains showed that the product yield of **6** in *P. crustosum* JZ54 at 0.26 ± 0.01 g per litre culture was comparable to 0.24 ± 0.02 g in BK08. In addition to the main metabolite **6**, its acetylated product annullatin G (**7**) was detected as a minor product in the cultures. In a previous report, it was speculated that the formation of **7** is catalysed by a host enzyme from *A. nidulans* (Xiang et al. 2022). Detailed analysis of the LC–MS chromatograms revealed that, with similar product yields of **6**, the conversion to **7** was three-fold higher in the *A. nidulans* strain BK08 than in JZ54 (Fig. 6).

## Discussion

Heterologous expression of genes and BGCs in a genetically well-studied host organism is regarded as a promising approach to unlock the vast microbial biosynthetic potential. In order to ensure the successful expression of genes originating from different species, it is essential to provide a diverse array of host organisms. In this study, we have expanded the repertoire of fungal heterologous expression systems by constructing a *P. crustosum* host strain, which can be used as an alternative to the commonly used model fungi *A. nidulans* (Chiang et al. 2016) and *A. oryzae* (Dao et al. 2021).

First, the DNA repair gene *ligD* was deleted to facilitate subsequent genetic manipulation in *P. crustosum*. Although a highly efficient *P. crustosum* Δ*ku70* strain had already been constructed prior to this study (Zhou and Li 2021), reports of telomere defects, reduced growth rates or increased sensitivity to UV exposure upon loss of *ku70* (Bugeja et al. 2012; Snoek et al. 2009; Meyer et al. 2007) have led to our decision to generate a Δ*ligD* strain to provide a genetically stable host for downstream applications. Subsequently, the biosynthetic pathways of the predominant metabolites terrestric acid and (hydroxy-)clavatol were inactivated to lower the endogenous SM background. The resulting Δ*traA*Δ*claF* mutant displayed a significantly reduced SM profile compared to the wild-type strain PRB-2. In particular, the formation of the highly reactive intermediate *ortho*-quinone methide was eliminated in *P. crustosum*. Previous studies have shown that *ortho-*quinone methide acts as a coupling agent for various natural product classes, including coumarins, flavonoids, cyclic dipeptides and indole derivatives (Wu et al. 2022; Liao et al. 2020). Although *ortho*-quinone methides are regarded as valuable reagents in synthetic chemical approaches to enhance the structural diversity of natural products (Singh et al. 2014), the presence in a host organism is rather undesirable as it impedes the identification and structural elucidation of heterologous products. Consequently, a clean SM background not only prevents unspecific conversions but also facilitates the detection and purification of novel molecules after heterologous gene expression, especially of minor products in the culture extracts. Moreover, the inactivation of highly active biosynthetic pathways allows for the reallocation of cellular resources and precursors towards the biosynthesis of heterologous compounds, thereby increasing the production yields (Pohl et al. 2020).

In order to provide an additional expression strain for constructs designed for the *A. nidulans* LO8030 model host, the commonly used *wA* expression site, consisting of the naphthopyrone synthase gene *wA* and its flanking regions, was integrated at the *P. crustosum pcr4401* locus. The resulting transformants exhibited a slight change in colouration and appeared pale green, indicating that *wA* can complement the function of *pcr4401* regarding spore pigment biosynthesis. In a previous study, the *pcr4401* gene locus was found to be a suitable expression site for heterologous genes (Kindinger et al. 2019). Similar to the *wA* integration site, transformants are easily detected by their white phenotype. To enable the use of constructs originally designed for *A. nidulans* also for *P. crustosum*, the *wA* gene including its flanking regions, which are required for homologous recombination-based gene expression, were integrated in lieu of *pcr4401*. By employing this strategy, the convenience of phenotype-based transformant identification remains unimpaired.

To further enhance the convenience of using *P. crustosum* as a heterologous expression host, the *pcribo* gene coding for the GTP cyclohydrolase II in the biosynthesis of riboflavin was deleted to provide an additional selection marker besides *pyrG* and the hygromycin phosphotransferase gene (*hph*) in *P. crustosum* JZ52. Although the *hph* selection marker has been successfully used for genetic manipulation of *P. crustosum* (Fan et al. 2019), its high toxicity represents a significant constraint in its broader application. In order to provide an alternative to the engineered *A. nidulans* host strain LO8030, which is auxotrophic for riboflavin, pyridoxine and uracil/uridine, the endogenous biosynthetic pathway of riboflavin was also inactivated in our *P. crustosum* uracil/uridine auxotrophic mutant. In conjunction with the *pyrG* marker recycling protocol established in a previous study (Zhou and Li 2021), our *P. crustosum* expression platform can be employed to conduct multiple genetic manipulation experiments in a single strain. A recent report described the construction of an updated *A.* *nidulans* host strain, designated LO11098, in which seven selection markers were incorporated (Lin et al. 2023). These findings highlight the potential for further optimization of our *P. crustosum* expression host.

The suitability of *P. crustosum* JZ52 as a host organism was demonstrated by the successful expression of a PKS gene and an entire BGC at the *P. crustosum wA* integration site using two *pyrG* and *afriboB*-based constructs initially designed for *A. nidulans*. As expected, the obtained transformants exhibited an albino phenotype, thus proving the functionality of the *wA* expression site. Moreover, the *A. nidulans gpdA* promoter was shown to drive gene expression also in *P. crustosum*, which is consistent with previous reports on the interchangeability of promoters and other genomic elements among different fungal species (Mózsik et al. 2021; Rantasalo et al. 2018; Hernanz-Koers et al. 2018). The use of *A. nidulans* constructs in our *P. crustosum* JZ52 host can prove beneficial in cases where products are further modified by endogenous enzymes from *A. nidulans*, as observed in a previous study (Xiang et al. 2020), or if homologous genes are present, thus hindering successful gene expression and the identification of new natural products. In addition, our data suggest that for the production of annullatin F, modification by endogenous host enzymes was reduced in *P. crustosum* JZ52 when compared to the commonly used *A. nidulans* LO8030. Although several well-established heterologous host systems are already available, these findings demonstrate the necessity and advantages of providing additional, alternative expression platforms.

In conclusion, we have constructed *P. crustosum* JZ52 as a promising expression system for fungal genes, especially those for SM production. Inactivation of the two predominant biosynthetic pathways resulted in a clean secondary metabolite profile in JZ52. To provide a valuable alternative to the *A. nidulans* LO8030 host, the *A. nidulans wA* site was used to replace the *P. crustosum pcr4401* pigment gene. Integration of the *wA* site allows for the use of constructs originally designed for *A. nidulans* also in *P. crustosum* and facilitates the detection of transformants based on their albino phenotype. To the best of our knowledge, the integration of a pigment-based expression site to enable the use of the same constructs in different fungal hosts was not reported prior to this study.

## Supplementary Information

Below is the link to the electronic supplementary material.

Supplementary file1 (PDF 1.76 KB)

## Data Availability

The authors declare that the data supporting the findings of this study are available within the paper and its Supplementary Information files. Should any raw data files be needed in another format, they are available from the corresponding author upon reasonable request.
