# Supplementary material for: Construction of an expression platform for fungal secondary metabolite biosynthesis in Penicillium crustosum
Source: Appl Microbiol Biotechnol. 2024 Jul 24;108(1):427. doi: 10.1007/s00253-024-13259-3 (PMC11269504; doi:10.1007/s00253-024-13259-3)
Supplement: Supplementary file 1 — Supplementary file1 (PDF 1.76 KB) [file 253_2024_13259_MOESM1_ESM.pdf]

SUPPORTING INFORMATION

Applied Microbiology and Biotechnology

Construction of an expression platform for fungal secondary metabolite biosynthesis in *Penicillium crustosum*

Jenny Zhou, Xiaoling Chen and Shu-Ming Li*

Institut für Pharmazeutische Biologie und Biotechnologie, Fachbereich Pharmazie, Philipps-Universität Marburg, Robert-Koch-Straße 4, 35037 Marburg, Germany

Corresponding Author

*Shu-Ming Li. Tel/Fax: + 49-6421-28-22461/25365

E-mail: shuming.li@staff.uni-marburg.de

ORCID Shu-Ming Li: 0000-0003-4583-2655

Table of contents

Table S1 *Aspergillus nidulans* strains used in this study S3

Table S2 Plasmids used and generated in this study S4

Table S3 Primers used in this study S6

Table S4 Spacer sequences targeting the *claF*, *pcr4401* and *pcribo* genes........ S10

Fig. S1 Schematic illustration of the Cas9 and gRNA expression plasmid pJZ66 .... S10

Fig. S2 BLASTp sequence alignment of Pcr2372 of *P. crustosum* with putative DNA ligase 4 of *Penicillium digitatum* S11

Fig. S3 BLASTp sequence alignment of Pcr2372 of *P. crustosum* with putative DNA ligase 4 of *Penicillium canariense* S12

Fig. S4 BLASTp sequence alignment of Pcr2372 of *P. crustosum* with putative DNA ligase 4 of *Penicillium brasilianum*........ S13

Fig. S5 Schematic illustration of *ligD* (*pcr2372*) deletion in *P. crustosum* FK15 and PCR verification of JZ03 (Δ*ligD*::*pyrG*) and JZ04 (Δ*ligD*Δ*pyrG*) by amplification of different partial fragments (P1–P4) from genomic DNA........ S14

Fig. S6 Schematic illustration of *traA* (*pcr11009*) deletion in *P. crustosum* JZ04 and PCR verification of JZ05 (Δ*traA*::*pyrG*) and JZ06 (Δ*traA*Δ*pyrG*) by amplification of different partial fragments (P1–P5) from genomic DNA........ S14

Fig. S7 BLASTn sequence alignment of 5S rRNA promoter of *P. crustosum* with 5S rRNA promoter sequence of *P. chrysogenum* deposited in the 5SRNAdb........ S15

Fig. S8 Schematic illustration of *claF* (*pcr3094*) deletion in *P. crustosum* JZ06 and PCR verification of JZ32 (Δ*claF*::*pyrG*) and JZ35 (Δ*claF*Δ*pyrG*) by amplification of different partial fragments (P1–P5) from genomic DNA. S15

Fig. S9 Schematic illustration of *wA* expression in *P. crustosum* JZ35 and PCR verification of JZ37 (*wA*::*pyrG*) and JZ38 (*wA*Δ*pyrG*) by amplification of different partial fragments (P1–P5) from genomic DNA. S16

Fig. S10 BLASTx sequence alignment of the gene product of *riboB* from *A. nidulans* with Pcr11223 from *P. crustosum*........ S16

Fig. S11 Schematic illustration of *pcribo* (*pcr11223*) deletion in *P. crustosum* JZ38 and PCR verification of JZ51 (Δ*pcribo*::*pyrG*) and JZ52 (Δ*pcribo*Δ*pyrG*) by amplification of different partial fragments (P1–P5) from genomic DNA. S17

Fig. S12 Schematic illustration of *orsA* expression in *P. crustosum* JZ38 and PCR verification of JZ39 (*gpdA*(p)::*pyrG*, empty vector control) and JZ40 (*gpdA*(*p*)-*orsA*::*pyrG*) by amplification of different partial fragments (P1–P2) from genomic DNA. S17

Fig. S13 Schematic illustration of annullatin (*anu*) cluster expression in *P. crustosum* JZ52 and PCR verification of JZ54 (*gpdA*(*p*)-*anuA*–*K*::*afriboB*) and JZ56 (*gpdA*(*p*)::*afriboB*, empty vector control) by amplification of downstream partial fragment (P1) from genomic DNA........ S18

References S19

Table S1 *Aspergillus nidulans* strains used in this study

Strain	Genotype	Source
LO8030	*pyroA4, riboB2, pyrG89, nkuA::argB,*<br>sterigmatocystin cluster (*AN7804 − AN7825*)Δ,<br>emericellamide cluster (*AN2545 − AN2549*)Δ,<br>asperfuranone cluster (*AN1039 − AN1029*)Δ,<br>monodictyphenone cluster (*AN10023 − AN10021*)Δ,<br>terrequinone cluster (*AN8512 − AN8520*)Δ,<br>austinol cluster part 1 (*AN8379 − AN8384*)Δ,<br>austinol cluster part 2 (*AN9246 − AN9259*)Δ,<br>F9775 cluster (*AN7906 − AN7915*)Δ,<br>asperthecin cluster (*AN6000 − AN6002*)Δ	(Chiang et al. 2016)
PX26	Δ*wA*::*gpdA(p)*-*orsA*::*pyrG* in LO8030	(Xiang and Li 2022)
BK08	Δ*wA*::*gpdA(p)*-*anuA*–*anuK*::*afriboB* in LO8030	(Xiang et al. 2022)

Table S2 Plasmids used and generated in this study

Plasmid	Description	Source
pESC-URA	*Saccharomyces cerevisiae* and *E. coli* shuttle vector	Agilent (Santa Clara, USA)
pFK23	*URA3*, *pcr4401* flanking, *A. nidulans gpdA* promoter, *A. fumigatus pyrG*, *ampR*	(Kindinger et al. 2019)
pCas9-tRp-gRNA	*Ustilaginoidea virens Gln-tRNA* promoter, gRNA spacer insert site, gRNA scaffold, *Aureobasidium pullulans* translation elongation factor (*Ptef*) promoter, *Cas9*, *Aspergillus awamori* glucoamylase terminator (*Tgla*), *ampR*	(Liang et al. 2018)
pYH-wA-pyrG	*URA3*, *wA* flanking, *gpdA*(*p*), *pyrG*, *ampR*	(Yin et al. 2013)
pPX26	*URA3*, *wA* flanking, *gpdA*(*p*), *orsA*, *pyrG*, *ampR*	(Xiang and Li 2022)
pJN017	*URA3*, *wA* flanking, *gpdA*(*p*), *afriboB*, *ampR*	(Kindinger et al. 2019)
pBK21	*URA3*, *wA* flanking, *gpdA*(*p*), *anuA–K*, *afriboB*, *ampR*	(Xiang et al. 2022)
pJZ03	Two-thirds of the *pyrG* marker at the 3'-end (1146 bps) originated from pFK23 were fused to the 1641 bps PCR fragment of the downstream region from *pcr2372* amplified from genomic DNA of *P. crustosum* PRB-2 and inserted into the *Bam*HI restriction site of the pESC-URA vector	This study
pJZ05	A 1575 bps PCR fragment of the upstream region from *pcr2372* amplified from genomic DNA of *P. crustosum* PRB-2 was fused to 358 bps of the downstream region, a 1872 bps PCR fragment including the *gpdA promoter* and two-thirds of the *pyrG* marker at the 5'-end originated from pFK23 and inserted into the *Bam*HI restriction site of the pESC-URA vector	This study
pJZ06	A 1189 bps PCR fragment of the upstream region from *pcr11009* amplified from genomic DNA of *P. crustosum* PRB-2 was fused to 327 bps of the downstream region, a 1145 bps PCR fragment of two-thirds of the *pyrG* marker at the 5'-end originated from pFK23 and inserted into the *Sma*I restriction site of the pESC-URA vector	This study
pJZ07	Two-thirds of the *pyrG* marker at the 3'-end (1140 bps) originated from pFK23 were fused to the 1527 bps PCR fragment of the downstream region from *pcr11009* amplified from genomic DNA of *P. crustosum* PRB-2 and inserted into the *Sma*I restriction site of the pESC-URA vector	This study
pJZ23	Two-thirds of the *pyrG* marker at the 3'-end (1140 bps) originated from pFK23 were fused to the 1643 bps PCR fragment of the downstream region from *pcr3094* amplified from genomic DNA of *P. crustosum* PRB-2 and inserted into the *Sma*I restriction site of the pESC-URA vector	This study
pJZ34	A 1534 bps PCR fragment of the upstream region from *pcr3094* amplified from genomic DNA of *P. crustosum* PRB-2 was fused to 328 bps of the downstream region, a 1145 bps PCR fragment of two-thirds of the *pyrG* marker at the 5'-end originated from pFK23 and inserted into the *Sma*I restriction site of the pESC-URA vector	This study

Table S2 Plasmids used and generated in this study (continued)

Plasmid	Description	Source
pJZ38	A 1043 bps PCR fragment of the upstream region from *pcr4401* amplified from genomic DNA of *P. crustosum* PRB-2 was fused to the 1000 bps *wA* upstream region and one-half of the *wA* gene (3642 bps), both amplified from genomic DNA of *A. nidulans* LO8030, a 1145 bps PCR fragment of two-thirds of the *pyrG* marker at the 5'-end originated from pFK23 and inserted into the *Sma*I restriction site of the pESC-URA vector	This study
pJZ39	Two-thirds of the *pyrG* marker at the 3'-end (1140 bps) originated from pFK23 was fused to one-half of the *A. nidulans wA* gene (3341 bps) and the 1000 bps *wA* downstream region, both amplified from genomic DNA of *A. nidulans* LO8030, a 1039 bps PCR fragment of the downstream region from *pcr4401* amplified from genomic DNA of *P. crustosum* PRB-2 and inserted into the *Sma*I restriction site of the pESC-URA vector	This study
pJZ65	A 175 bps PCR fragment of the 5S rRNA amplified from genomic DNA of *P. crustosum* PRB-2 was cloned into the *Kpn*I and *Bam*HI restriction site of the pmCas9-tRp-gRNA vector	This study
pJZ66	The gRNA spacer sequence AAGCCATCTATACGACAGCTGGG designed for the deletion of *pcr3094* was annealed using corresponding sense and antisense oligonucleotides and cloned into the *BsmB*I restriction site of pJZ65	This study
pJZ85	The 23 bps gRNA spacer sequence TGGGAACAGAGTGCATGTTGTGG designed for the expression of the *A. nidulans wA* gene in the *P. crustosum pcr4401* locus was annealed using corresponding sense and antisense oligonucleotides and cloned into the *BsmB*I restriction site of pJZ65	This study
pJZ96	A 1579 bps PCR fragment of the upstream region from *pcr11223* amplified from genomic DNA of *P. crustosum* PRB-2 was fused to 406 bps of the downstream region, a 1145 bps PCR fragment of two-thirds of the *pyrG* marker at the 5'-end originated from pFK23 and inserted into the *Sma*I restriction site of the pESC-URA vector	This study
pJZ97	Two-thirds of the *pyrG* marker at the 3'-end (1140 bps) originated from pFK23 were fused to the 1576 bps PCR fragment of the downstream region from *pcr11223* amplified from genomic DNA of *P. crustosum* PRB-2 and inserted into the *Sma*I restriction site of the pESC-URA vector	
pJZ98	The 23 bps gRNA spacer sequence TACCTGCGTCAGGAGGGTCGTGG designed for the deletion of *pcr11223* was annealed using corresponding sense and antisense oligonucleotides and cloned into the *BsmB*I restriction site of pJZ65	This study

Table S3 Primers used in this study

Primer	Oligonucleotide sequence 5’-3’	Function
gdpApyrGr1	CGGCCGCATTCTGTCTGAGAG	Amplification of 2/3 from the *pyrG* gene (3'end) for split marker cloning in pJZ03
pyrG_pESCf1	TTAATATACCTCTATACTTTAACGTCAAGAACCCGAGAACTCCTGGACC	
3UTR_pyrGf1	CAGTGCCTCCTCTCAGACAGAATGCGGCCGCTGAACCGCAAGAGCCGAG	Amplification of the downstream region of *pcr2372* from *P. crustosum*
3UTR_pESCr1	GGGCCCTATAGTGAGTCGTATTACGGATCGCGAAAGGCAATGATCGAC	
5UTR_pESCf1	AATATACCTCTATACTTTAACGTCAAGGAGGTTGGACATAAGGTTCAC	Amplification of the upstream region of *pcr2372* from *P. crustosum*
5UTR_300br1	CACGATTGAACTCGGCTCTTGCGGTTCAGTTTGGAGAAGTGAAGAAC	
300b_5UTRf1	CCAACTTTTGGTTCTTCACTTCTCCAAACTGAACCGCAAGAGCCGAG	Amplification of 358 bps of the downstream region of *pcr2372* from *P. crustosum* for *pyrG* marker recycling
300b_gdpAr1	CTGCGACCGTCCGTCTCTCCGCATGTATGGCCATGGCACGAGATCAC	
gdpApyrGf1	CATGCGGAGAGACGGACG	Amplification of 2/3 from the *pyrG* gene (5'end) for split marker cloning in pJZ05
pyrG_pESCr1	GGGCCCTATAGTGAGTCGTATTACGGATCCATCCTCCGAGGCTGAAGAC	
5ligDver_f2	TGCGATGATGAGCTTGTGC	Screening of Δ*pcr2372* transformants
pyrGver_r1	GCTCCATATTCTCCGATGATG	
pyrGver_f1	GAGGAAGGCTGCATACATTG	
3ligDver_r2	GGTCGACATAAATGTGGAATGC	
JZ05_5pyrG_f	GCTAGCGAGAGTTATTCTGTGTCTG	Screening of all Δ*pyrG* transformants
		Amplification of 2/3 from the *pyrG* gene (5'end) for split marker cloning
JZ08_3pyrG_r	GCGGCCGCATTCTGTCTG	Screening of all Δ*pyrG* transformants
		Amplification of 2/3 from the *pyrG* gene (3'end) for split marker cloning
JZ06_5pyrG_r	GAAATCAACTTCTGTTCCATGTCGACGCCCGAGGCTGAAGACACATCCG	Amplification of 2/3 from the *pyrG* gene (5'end) for split marker cloning
JZ07_3pyrG_f	GATCCGTAATACGACTCACTATAGGGCCCGAACTCCTGGACCTCGCTG	Amplification of 2/3 from the *pyrG* gene (3'end) for split marker cloning

Table S3 Primers used in this study (continued)

JZ01_traAup_f	CCGGATCCGTAATACGACTCACTATAGGGCCCGGGCCTTTGATGGCAGG	Amplification of the upstream region of *pcr11009* from *P. crustosum*
JZ02_traAup_r	GTGAAATTCTCTAACTATGGTGCTGTAAGCAATAGCAATGGGCTCGG	
JZ03_traA300d_f	CTTACAGCACCATAGTTAGAG	Amplification of 327 bps of the downstream region of *pcr11009* from *P. crustosum* for *pyrG* marker recycling
JZ04_traA300d_r	TCAGACACAGAATAACTCTCGCTAGCGGTACGACAATGTATGGTAAATC	
JZ09_traAdown_f	TGCCTCCTCTCAGACAGAATGCGGCCGCCTTACAGCACCATAGTTAGAG	Amplification of the downstream region of *pcr11009* from *P. crustosum*
JZ10_traAdown_r	AAATCAACTTCTGTTCCATGTCGACGCCCCAACTGCCGCTCCATAG	
JZ36_pyrGver1	CTATTGGACGCGGTGCCGACTTTATCATCG	Screening of Δ*pcr11009* transformants
		Screening of Δ*pcr3094* transformants
		Screening of *gpdA*::*pyrG* transformants
		Screening of *gpdA*::*orsA*::*pyrG* transformants
		Screening of Δ*pcr11223* transformants
JZ37_pyrG_ver2	GAGACAGGCCACATCGGTGCTGTATTCCTC	Screening of Δ*pcr11009* transformants
		Screening of Δ*pcr3094* transformants
		Screening of *gpdA*::*pyrG* transformants
		Screening of *gpdA*::*orsA*::*pyrG* transformants
		Screening of Δ*pcr11223* transformants
JZ38_traAdo_v_r2	CGGAGATCCATTACTCGGCTTGACATACCAC	Screening of Δ*pcr11009* transformants
JZ63_traAdo_v_r	TGGCAGCCGCTCTGCAGG	
JZ43_traAscr_f	GAACAGGATGCGAGCACTATCTGCACCATC	
JZ44_traAscr_r	CTTTCCAGCAATCACGGTTCTGGTTCCTAG	
JZ68_claF_3F_R	AAATCAACTTCTGTTCCATGTCGACGCCCCGCTGGCTGTGGCATTCTC	Amplification of the downstream region of *pcr3094* from *P. crustosum*
JZ69_claF_3F_F	GTGCCTCCTCTCAGACAGAATGCGGCCGCGGAGCATCGGCTTGTTTG	Amplification of the downstream region of *pcr3094* from *P. crustosum*
JZ126_claF_5F_f	GATCCGTAATACGACTCACTATAGGGCCCCGCCTTGTATTCGCCCAAG	Amplification of the upstream region of *pcr3094* from *P. crustosum*
JZ127_claF_5F_r	GATAACATTAATCAAACAAGCCGATGCTCCGTTGCTAGTCGCGTTGAGG	

Table S3 Primers used in this study (continued)

JZ66_claF_300b_F	GGAGCATCGGCTTGTTTG	Amplification of 328 bps of the downstream region of *pcr3094* from *P. crustosum* for *pyrG* marker recycling
JZ67_claF_300b_R	TTCGTCAGACACAGAATAACTCTCGCTAGCATC GGCCGCTTATACGG	
JZ190_RNAclaF1_f	ATGTAAGCCATCTATACGACAGCTGGG	sense and antisense oligonucleotides for the annealing of the gRNA spacer designed for the deletion of *pcr3094*
JZ191_RNAclaF_r	AAACCCCAGCTGTCGTATAGATGGCTT	
JZ100_claF_5F_R2	GATAACATTAATCAAACAAGCCGATGCTCCACC GGTAACGAGCACACAC	Screening of Δ*pcr3094* transformants
JZ117_claF_5F_V	GGCCATGGAACTGGTTCGAGAAGTCG	
JZ118_claF_3F_V	CTCGAGCTGGCTGCAGTGCC	
JZ161_claF_5F_V	CGGGTATAGCTGCAATCGGGCC	
JZ70_4401up_pESC	TATGGTAACCCAGCTGGAACC	Amplification of the upstream region of *pcr4401* from *P. crustosum*
JZ125_4401up_f	GATCCGTAATACGACTCACTATAGGGCCCGC TTGAGAACATTTGGGTCG	
JZ139_wA1_f	TTTCCCAATGGTTCCAGCTGGGTTACCATAGC TCTGGAACAGTCTCGCC	Amplification of upstream flanking region and 1/2 of the *wA* gene (5'end) from *A. nidulans*
JZ140_wA1_r	CAGACACAGAATAACTCTCGCTAGCGATTAC CGGCAGTGTCTTACCAGG	
JZ141_wA2_f	CAGTGCCTCCTCTCAGACAGAATGCGGCCGC AGCTCTTGAACGAGAAGG	Amplification of downstream flanking region 1/2 of the *wA* gene (3'end) from *A. nidulans*
JZ142_wA2_r	CTGCTGTCAGTACGCGAAG	
JZ143_4401d_f	CTGGAGGAGATCTTCGCGTACTGACAGCAGC ATTGAACACCTCCCAGCC	Amplification of the downstream region of *pcr4401* from *P. crustosum*
JZ144_4401d_r	AAATCAACTTCTGTTCCATGTCGACGCCCCG GTGGTAGTTCTGCTGAAC	
JZ234_RNA4401_2f	ATGTTGGGAACAGAGTGCATGTTGTGG	sense and antisense oligonucleotides for the annealing of the gRNA spacer designed for the expression of *wA* in the *P. crustosum pcr4401* locus
JZ235_RNA4401_2r	AAACCCACAACATGCACTCTGTTCCCA	
JZ128_wA1_r	GGCGGAAGATGAGAGATCTC	Screening of Δ*pcr4401*::*wA* transformants
JZ75_wAdown_f	CCGAGTTTGGCGTATACTAC	
JZ123_wA_Fra2_r	CCTGAGCCTTTGAGCTCTGGC	
JZ133_wA4_f	GCTAGCGATGGAACAGACTC	
JZ177_4401ver_f	CGATTGGCCATGCGAGG	Screening of Δ*pcr4401*::*wA* transformants
		Screening of *gpdA*(p)::*pyrG* transformants
		Screening of *gpdA*(p)-*orsA*::*pyrG* transformants
JZ178_4401ver_r	GCTCAGAAACGCACACTGG	Screening of Δ*pcr4401*::*wA* transformants
		Screening of *gpdA*(p)::*pyrG* transformants
		Screening of *gpdA*(p)-*orsA*::*pyrG* transformants

Table S3 Primers used in this study (continued)

JZ188_5SsRNA_f	ACGACTCACTATAGGGCGAATTGGGTACCAC ATACGACCATAGGGTGTG	Amplification of the 5S rRNA promoter from *P. crustosum*
JZ189_5SsRNA_r	AACCGAGACGCTGGATCCGACGTCTCGACAT ACAACAGTAGGGATTCGC	
JZ243_pPX26ver_r	CCGTGCTTTCTGTCATGACC	Screening of *gpdA*(*p*)-*orsA*::*pyrG* transformants
JZ257_5FRibo_f	AAAAACCCCGGATCCGTAATACGACTCACTA TAGGGCCCAAGATCGAGGAAGGGCCGTC	Amplification of the upstream region of *pcr11223* from *P. crustosum*
JZ258_5FRibo_r	GATGATGGGGGCAAAAGTTCAAAACGACAA GCTCCAGGGATGCGACGGCGGAGAGG	
JZ259_300bRibo_f	TCCCTGGAGCTTGTCGTTTTG	Amplification of 406 bps of the downstream region of *pcr11223* from *P. crustosum* for *pyrG* marker recycling
JZ260_300bRibo_r	TTCGTCAGACACAGAATAACTCTCGCTAGCC GTGCGGCAATGGGAATTATC	
JZ261_3FRibo_f	CACGCATCAGTGCCTCCTCTCAGACAGAATG CGGCCGCTCCCTGGAGCTTGTCGTTTTG	Amplification of the downstream region of *pcr11223* from *P. crustosum*
JZ262_3FRibo_r	CTTCTTCGGAAATCAACTTCTGTTCCATGTCG ACGCCCCGCTGGTAAGTTTTGTATCGG	
JZ263_sgRNARibof	ATGTTACCTGCGTCAGGAGGGTCGTGG	sense and antisense oligonucleotides for the annealing of the gRNA spacer designed for the deletion of *pcr11223*
JZ264_sgRNARibor	AAACCCACGACCCTCCTGACGCAGGTA	
JZ267_ribo5V_f	CCGCCAAAGCCAGAAGCTCAGC	Screening of Δ*pcr11223* transformants
JZ268_ribo3V_r	GATCCTGTCACGCATTGCCGGCC	
JZ269_riboGen_f	ACCGGCCCACGCCTCACC	
JZ270_riboGen_r	CCTTTCTGGCCACCAGTCCGC	
JZ57_An_traB_5Vr	CCTCTCTAACCTCTGGTTCGC	Screening of *gpdA*(p)::*afriboB* transformants
		Screening of *gpdA*(p)-*anuA–K*::*afriboB* transformants
JZ59_An_traB_GVr	CTCATGCATTCAGCGAGAGGG	Screening of *gpdA*(p)::*afriboB* transformants
		Screening of *gpdA*(p)-*anuA–K*::*afriboB* transformants

Table S4 Spacer sequences targeting the *claF*, *pcr4401* and *pcribo* genes

Target	gRNA	PAM
claF	AAGCCATCTATACGACAGCT	GGG
pcr4401	TGGGAACAGAGTGCATGTTG	TGG
pcribo	TACCTGCGTCAGGAGGGTCG	TGG

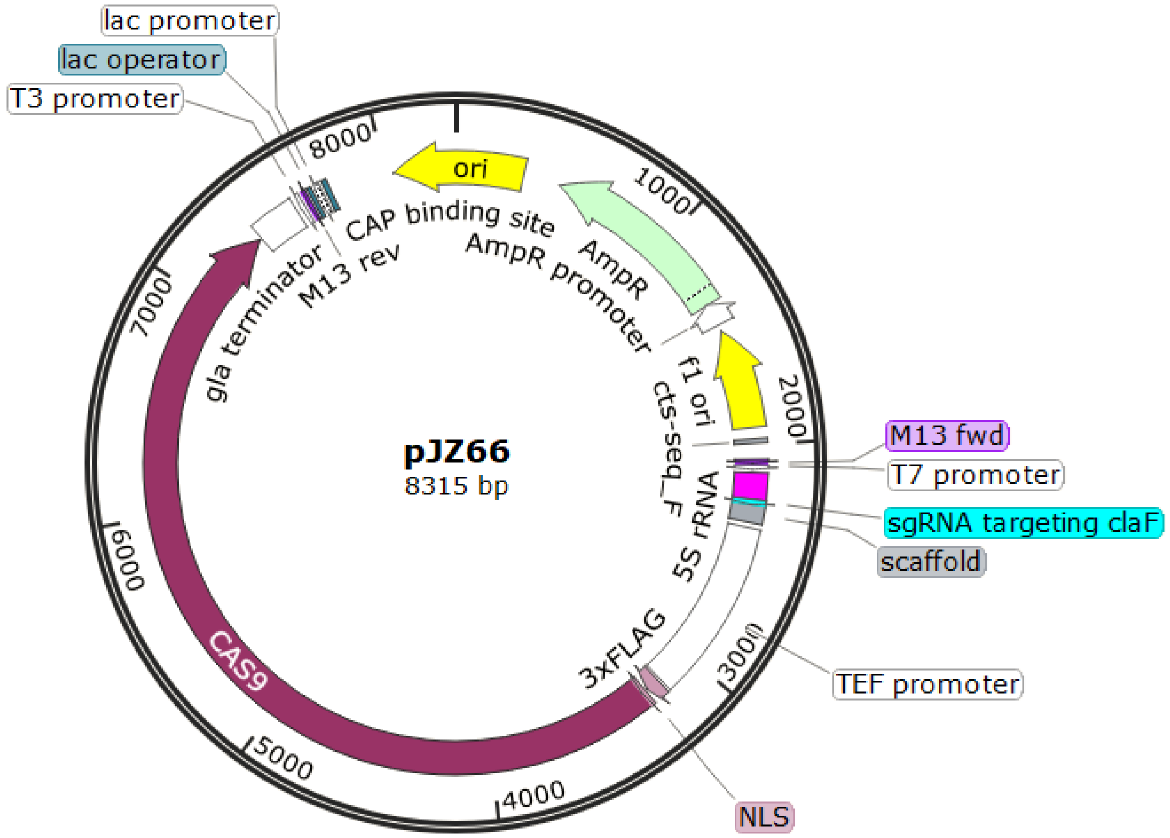

Fig. S1 Schematic illustration of the Cas9 and gRNA expression plasmid pJZ66 (gRNA: guide RNA, PAM: protospacer adjacent motif, *TEF* promoter: *Aureobasidium pullulans* translation elongation factor promoter, NLS: nuclear localization signal, *gla* terminator: *Aspergillus awamori* glucoamylase terminator, *lac*: lactose operon, ori: origin of replication, AmpR: ampicillin resistance)

DNA ligase 4 [Penicillium digitatum]

Sequence ID: **XP_065956922.1** Length: 1000 Number of Matches: 1

Range 1: 2 to 969

Score	Expect	Method	Identities	Positives	Gaps	Frame
1811 bits(4691)	0.0()	Compositional matrix adjust.	883/968(91%)	916/968(94%)	8/968(0%)	

```
Query  1    DSDEILGQDLPKLENEEDLDEK--------APTLPFHDLYLNLFNPLSELKKKPSGPAPA  52
            DSDEIL Q+L   E EED+  K        APTLPFH+LYLNL NPLSELKKK SGPAPA
Sbjct  2    DSDEILSQELLGQEKEEDIKAKYPNRPHNRAPTLPFHELYLNLLNPLSELKKKLSGPAPA  61

Query  53   RRKVGPHGKGATSLNPFERRRDVIERFISRWRKDVGDDIYPALRLILPDKDRDRPMYGIK  112
            RRKVGP+GKGA SLNPFERRRDVIERFISRWRKDVG+DIYPALRLILPDKDRDRPMYGIK
Sbjct  62   RRKVGPNGKGAASLNPFERRRDVIERFISRWRKDVGNDIYPALRLILPDKDRDRPMYGIK  121

Query  113  EKAIGKMLVKIMKINKESEDGYNLLNWKLPGQGTTTRMAGDFAGRCFDVLSKRPMRTEPG  172
            EKAIGKMLVKIMKINKESEDGYNLLNWKLPGQG TTRMAGDFAGRCFDVLSKRPMRTEPG
Sbjct  122  EKAIGKMLVKIMKINKESEDGYNLLNWKLPGQGATTRMAGDFAGRCFDVLSKRPMRTEPG  181

Query  173  DMTIDEVNEKLDKLSAASKEDEQLPILTEFYRRMNPEELLWLIRIILRQMKVGATERTLF  232
            DMTIDEVNEKLDKLSAASKEDEQLPILTEFYRRMNPEELLWL+RIILRQMKVGATERTLF
Sbjct  182  DMTIDEVNEKLDKLSAASKEDEQLPILTEFYRRMNPEELLWLVRIILRQMKVGATERTLF  241

Query  233  DVWHPDAENLYSISSSLRRVCWELHDPNIRLEGEERGIALMQCFQPQLAQFQMHSFEKII  292
            DVWHPDAENLYSISSSLRRVCWELHDPNIRLEGEERGIALMQCFQPQLAQFQMHSFEKII
Sbjct  242  DVWHPDAENLYSISSSLRRVCWELHDPNIRLEGEERGIALMQCFQPQLAQFQMHSFEKII  301

Query  293  ARMKPTEDDNVFWIEEKMDGERMQLHMAPDDSTKGGRKFGFWSRKAKEYTYLYGNGICDE  352
            ARMKPTEDD+VFWIEEKMDGERMQLHMAPDDS +GGRKFGFWSRKAKEYTYLYGNGI DE
Sbjct  302  ARMKPTEDDHVFWIEEKMDGERMQLHMAPDDSIQGGRKFGFWSRKAKEYTYLYGNGIYDE  361

Query  353  NGALTRHLKDAFVDGVQSIILDGEMITWDPEQDAIVPFGTLKTAALAEQRNPFSNGPRPL  412
            NGALTRHLKDAFVDGVQSIILDGEMITWDPEQDA+VPFGTLKTAALAEQRNPFSN PRPL
Sbjct  362  NGALTRHLKDAFVDGVQSIILDGEMITWDPEQDAMVPFGTLKTAALAEQRNPFSNAPRPL  421

Query  413  FRVFDILHLNGRDLTKYTLRDRRNALEKTVRPVHRRFEIHSYEEATTTTEVEAALRKVVA  472
            FRVFDILHLNGRDLTKY LRDRRNALEKT+RPV+RRFEIHSYEEATTTTEVE ALRKVVA
Sbjct  422  FRVFDILHLNGRDLTKYALRDRRNALEKTIRPVYRRFEIHSYEEATTTTEVEKALRKVVA  481

Query  473  EASEGLVLKNPRSPYRLNERHDDWMKVKPDYMTEFGESLDVVVIGGYYGSGHRGGALSSF  532
            EASEGLVLKNPRSPYRLNERHDDWMKVKPDYMTEFGESLDVVVIGGYYGSGHRGGALSSF
Sbjct  482  EASEGLVLKNPRSPYRLNERHDDWMKVKPDYMTEFGESLDVVVIGGYYGSGHRGGALSSF  541

Query  533  LCGLRVDDSTQAAEKCWSFCKVGGGFTAADYQEVRHHTDGKWKAWDAKKPPTTFIELAGG  592
            LCGLRVDDSTQAAEKCWSFC+VGGGFTAADYQEVRHHT+GKWK WDAKKPPT FIELAGG
Sbjct  542  LCGLRVDDSTQAAEKCWSFCRVGGGFTAADYQEVRHHTEGKWKVWDAKKPPTNFIELAGG  601

Query  593  DAQHERPDMWIKPSDSIVLCVKAASVAISDQFRMGLTLRFPRFKKLRKDKDWKSALSVQE  652
            DAQHERPDMWIKPSDSIVLC KAASVAISDQFRMGLTLRFPRFKKLRKDKDWKSALSVQE
Sbjct  602  DAQHERPDMWIKPSDSIVLCAKAASVAISDQFRMGLTLRFPRFKKLRKDKDWKSALSVQE  661

Query  653  FLDLKSNAEQEHREKEFSVDNSRTKRVKRTTKKPLTVAGYDDNIDVQYLGPSGHVFDELN  712
            FLDLKSNAEQEHREKEFSVDNSR KRVKR TKKPLTVAGYDDNIDVQYL PSGH+FDELN
Sbjct  662  FLDLKSNAEQEHREKEFSVDNSRKKRVKRATKKPLTVAGYDDNIDVQYLEPSGHIFDELN  721

Query  713  FFIMTESTIPEKKTKPQLEQLVKANGGKIYQTRTAAVDTLCIAERRTVKVASLQKSGEQS  772
            FF+MTEST PEKKTK QLEQLVKANGGKIYQT+TAAVDTLC+A+RRTVKVASLQ+SGEQ+
Sbjct  722  FFVMTESTSPEKKTKLQLEQLVKANGGKIYQTKTAAVDTLCVADRRTVKVASLQRSGEQN  781

Query  773  IIRPSWLIDCVKQNEIDAGLPDLLLPFEPRHMFFMTEDKEEEVAANVDKFMDSYARDTTV  832
            IIRPSWLIDC+KQNEID GLPDLLLPFEPRHMFFM ED+EEEV ANVD+FMDSYARDTTV
Sbjct  782  IIRPSWLIDCIKQNEIDIGLPDLLLPFEPRHMFFMREDEEEEVVANVDQFMDSYARDTTV  841

Query  833  DELKDIFNQMEQNQKQFDHAPDSETIQRVEARIQEKVNAGYTVPCGWLFRGLKFYFYSNK  892
            +ELK++F QMEQNQ+Q DHA D ETIQRVEARIQEK+NAGYTVPCGWLFRGLKFYF+SN
Sbjct  842  EELKEVFKQMEQNQEQPDHALDPETIQRVEARIQEKINAGYTVPCGWLFRGLKFYFHSNG  901

Query  893  DHPDEPTSREPRKEDQRLQFARNTARFAGAESASSFKSSGTTHVIVDPDNLSSADISSLR  952
            D  DE  S+E  K+ Q L  ARNTARFAGAESASS KSSGTTHVIVDP+ LSSADISSLR
Sbjct  902  DRQDESASQELWKKSQPLYLARNTARFAGAESASSLKSSGTTHVIVDPETLSSADISSLR  961

Query  953  KSLAERPG  960
            KSLAE+PG
Sbjct  962  KSLAEKPG  969
```

Fig. S2 BLASTp sequence alignment of Pcr2372 of *P. crustosum* (Query) with putative DNA ligase 4 of *Penicillium digitatum* (XP_065956922)

DNA ligase 4 [Penicillium canariense]

Sequence ID: **XP_056541564.1** Length: 1006 Number of Matches: 1

Range 1: 2 to 975

Score	Expect	Method	Identities	Positives	Gaps	Frame
1575 bits(4078)	0.0()	Compositional matrix adjust.	758/977(78%)	849/977(86%)	20/977(2%)	

```
Query  1    DSDEILGQDLPKLENEEDLDEK--------APTLPFHDLYLNLFNPLSELKKKPSGPAPA  52
            DSDEI+ +  P    EEDLDEK        APT PFH+LYLNLFNPLS++KKKP GP+ A
Sbjct  2    DSDEIIPE--PTQNEEEDLDEKYPNRPHNRAPTFPFHELYLNLFNPLSDIKKKPKGPSLA  59

Query  53   RRKVGPHGKGATSLNPFERRRDVIERFISRWRKDVGDDIYPALRLILPDKDRDRPMYGIK  112
            RRK GP GK   SLNP+E RRD+I RFISRWRK+VGDDIYPA RLILPDKDRDRPMYGIK
Sbjct  60   RRKAGPQGKSTASLNPYELRRDIIARFISRWRKEVGDDIYPAFRLILPDKDRDRPMYGIK  119

Query  113  EKAIGKMLVKIMKINKESEDGYNLLNWKLPGQGTTTRMAGDFAGRCFDVLSKRPMRTEPG  172
            EK IGKMLVKIMKINKESED  NLLNWKLPG  +  RMAGDFAGRC+DVLSKRPMRTEPG
Sbjct  120  EKVIGKMLVKIMKINKESEDASNLLNWKLPGV-SAARMAGDFAGRCYDVLSKRPMRTEPG  178

Query  173  DMTIDEVNEKLDKLSAASKEDEQLPILTEFYRRMNPEELLWLIRIILRQMKVGATERTLF  232
            DM+I+EVN+KLD LSAASKED+Q PILTEFYRRMNP+EL WLIRIILRQMKVGATERTLF
Sbjct  179  DMSIEEVNKKLDDLSAASKEDQQTPILTEFYRRMNPDELTWLIRIILRQMKVGATERTLF  238

Query  233  DVWHPDAENLYSISSSLRRVCWELHDPNIRLEGEERGIALMQCFQPQLAQFQMHSFEKII  292
            +VWHPDAENLYSISSSLRRVCWELHDPNIRLE  +RGI+LMQCFQPQLAQFQMHSFEK+I
Sbjct  239  NVWHPDAENLYSISSSLRRVCWELHDPNIRLEAGDRGISLMQCFQPQLAQFQMHSFEKMI  298

Query  293  ARMKPTEDDNVFWIEEKMDGERMQLHMAPDDSTKGGRKFGFWSRKAKEYTYLYGNGICDE  352
            +RM+PTEDD VFWIEEKMDGERMQLHM  D+S  GGR+F FWSRKAKEYTYLYG+GI DE
Sbjct  299  SRMQPTEDDPVFWIEEKMDGERMQLHMDQDESVPGGRRFCFWSRKAKEYTYLYGSGIYDE  358

Query  353  NGALTRHLKDAFVDGVQSIILDGEMITWDPEQDAIVPFGTLKTAALAEQRNPFSNGPRPL  412
             G LTRHLKDAFVDGVQ++ILDGEM+TWDP+QDA VPFGTLKTAALAEQRNPFS+GPRPL
Sbjct  359  KGTLTRHLKDAFVDGVQNLILDGEMVTWDPKQDAPVPFGTLKTAALAEQRNPFSDGPRPL  418

Query  413  FRVFDILHLNGRDLTKYTLRDRRNALEKTVRPVHRRFEIHSYEEATTTTEVEAALRKVVA  472
            FRVFDIL+LNGRDLT+YTLRDRRNAL+KTV PVHRRFEI  Y EATT  +VE  LR VVA
Sbjct  419  FRVFDILYLNGRDLTRYTLRDRRNALQKTVLPVHRRFEILEYHEATTAADVEKRLRTVVA  478

Query  473  EASEGLVLKNPRSPYRLNERHDDWMKVKPDYMTEFGESLDVVVIGGYYGSGHRGGALSSF  532
            EASEGLVLKNPRSPYRLNERHDDWMKVKP+YMTEFGESLD+VVIGGYYGSGHRGGAL+SF
Sbjct  479  EASEGLVLKNPRSPYRLNERHDDWMKVKPEYMTEFGESLDLVVIGGYYGSGHRGGALASF  538

Query  533  LCGLRVDD--STQAAE--KCWSFCKVGGGFTAADYQEVRHHTDGKWKAWDAKKPPTTFIE  588
            LCGLRVDD  S+Q A+  KCWSFCKVGGGFTAADYQE+RHHTDGKWK WD+KKPPT  IE
Sbjct  539  LCGLRVDDQNSSQGADPMKCWSFCKVGGGFTAADYQEIRHHTDGKWKEWDSKKPPTALIE  598

Query  589  LAGGDAQHERPDMWIKPSDSIVLCVKAASVAISDQFRMGLTLRFPRFKKLRKDKDWKSAL  648
            LAGGDAQ+ERPDMWIKPSDS+VLCVKAASV++SDQFRMGLTLRFPRFKKLRKDK WK+AL
Sbjct  599  LAGGDAQYERPDMWIKPSDSVVLCVKAASVSVSDQFRMGLTLRFPRFKKLRKDKTWKTAL  658

Query  649  SVQEFLDLKSNAEQEHREKEFSVDNSR-TKRVKRTTKKPLTVAGYDDNIDVQYLGPSGHV  707
            S+QEFLDLKSNAEQEHREKEFSVDNSR  KRVK+T KKPLT+AGY++  + QYLGPSGHV
Sbjct  659  SIQEFLDLKSNAEQEHREKEFSVDNSRKQKRVKKTGKKPLTIAGYEEKENAQYLGPSGHV  718

Query  708  FDELNFFIMTESTIPEKKTKPQLEQLVKANGGKIYQTRTAAVDTLCIAERRTVKVASLQK  767
            FD LNFF+MTEST+PEKKTK +LEQLVKANGGKIYQT TAA DT+CIA+R+TVKVAS+QK
Sbjct  719  FDHLNFFVMTESTMPEKKTKAELEQLVKANGGKIYQTNTAAPDTICIADRKTVKVASIQK  778

Query  768  SGEQSIIRPSWLIDCVKQNEIDAGLPDLLLPFEPRHMFFMTEDKEEEVAANVDKFMDSYA  827
            SG+ +I+RPSWLIDCVKQNE DAGLPDLLL FEPRHMFF+ ED++E++  NVD+F DSYA
Sbjct  779  SGQTNIVRPSWLIDCVKQNEKDAGLPDLLLAFEPRHMFFIKEDQDEDIKRNVDQFGDSYA  838

Query  828  RDTTVDELKDIFNQMEQNQKQFDHAPDSETIQRVEARIQEKVNAGYTVPCGWLFRGLKFY  887
            RDT+VDEL+DI  QM+  + + + + D  ++ ++EA IQ+K+N+GYT PCGWLFRGL+F
Sbjct  839  RDTSVDELEDILQQMQHGKDEVESSLDPHSVHKIEAHIQDKINSGYTAPCGWLFRGLQFL  898

Query  888  FY----SNKDHPDEPTSREPRKEDQRLQFARNTARFAGAESASSFKSSGTTHVIVDPDNL  943
            F+    +N +   E  S     ED RL  ARNTA FAGA S +S  S   THVIV+P+ +
Sbjct  899  FFPTHQANGNESGESDSSGSITEDIRLTLARNTAHFAGASSVTSLNSPDVTHVIVNPEKV  958

Query  944  SSADISSLRKSLAERPG  960
            SSADI SLR+SL  R G
Sbjct  959  SSADIRSLRQSLVARSG  975
```

Fig. S3 BLASTp sequence alignment of Pcr2372 of *P. crustosum* (Query) with putative DNA ligase 4 of *Penicillium canariense* (XP_056541564)

DNA ligase 4 [Penicillium brasilianum]

Sequence ID: **OOQ89364.1** Length: 1006 Number of Matches: 1

Range 1: 15 to 971

Score	Expect	Method	Identities	Positives	Gaps	Frame
1558 bits(4034)	0.0()	Compositional matrix adjust.	743/958(78%)	834/958(87%)	18/958(1%)	

```
Query  16    EEDLDEK--------APTLPFHDLYLNLFNPLSELKKKPSGPAPARRKVGPHGKGATSLN  67
             EED DEK        APTLPFHDLYLNLF+PLS +KK+P     ARRK GPHGKGA SLN
Sbjct  15    EEDFDEKYPNRPHNSAPTLPFHDLYLNLFDPLSGIKKRPKASGLARRKAGPHGKGAESLN  74

Query  68    PFERRRDVIERFISRWRKDVGDDIYPALRLILPDKDRDRPMYGIKEKAIGKMLVKIMKIN  127
             P+E RRD+I RFISRWRK+VGDDIYPA RLILPDKDRDRPMYG+KEK IGKMLVKIMKIN
Sbjct  75    PYELRRDIIARFISRWRKEVGDDIYPAFRLILPDKDRDRPMYGMKEKVIGKMLVKIMKIN  134

Query  128   KESEDGYNLLNWKLPGQGTTTRMAGDFAGRCFDVLSKRPMRTEPGDMTIDEVNEKLDKLS  187
             KESED  NLLNWKLPG  +  RMAGDFAGRC+DV+SKRPMRTEPGDMTI+EVN+KLD LS
Sbjct  135   KESEDASNLLNWKLPGV-SAARMAGDFAGRCYDVISKRPMRTEPGDMTIEEVNKKLDDLS  193

Query  188   AASKEDEQLPILTEFYRRMNPEELLWLIRIILRQMKVGATERTLFDVWHPDAENLYSISS  247
             AASKED+Q+PIL EFYRRMNP+EL WLIRIILRQMKVGATERTLF+VWHPDAENLYSISS
Sbjct  194   AASKEDQQVPILAEFYRRMNPDELTWLIRIILRQMKVGATERTLFNVWHPDAENLYSISS  253

Query  248   SLRRVCWELHDPNIRLEGEERGIALMQCFQPQLAQFQMHSFEKIIARMKPTEDDNVFWIE  307
             SLRRVCWELHDPNIRLE E+RGI LMQCFQPQLAQFQMHSFEK+I+RM+PTE+D VFWIE
Sbjct  254   SLRRVCWELHDPNIRLEAEDRGIGLMQCFQPQLAQFQMHSFEKMISRMRPTEEDPVFWIE  313

Query  308   EKMDGERMQLHMAPDDSTKGGRKFGFWSRKAKEYTYLYGNGICDENGALTRHLKDAFVDG  367
             EKMDGERMQLHMAPD+S  GGR+F FWSRKAKEYTYLYG+GI DE GALT+HL++AF DG
Sbjct  314   EKMDGERMQLHMAPDESIPGGRRFRFWSRKAKEYTYLYGSGIYDEKGALTKHLENAFADG  373

Query  368   VQSIILDGEMITWDPEQDAIVPFGTLKTAALAEQRNPFSNGPRPLFRVFDILHLNGRDLT  427
             VQS+ILDGEM+TWDP+QDA VPFGTLKTAALAEQRNPFS+GPRPLFRVFDIL+LNG DLT
Sbjct  374   VQSLILDGEMVTWDPKQDAPVPFGTLKTAALAEQRNPFSDGPRPLFRVFDILYLNGTDLT  433

Query  428   KYTLRDRRNALEKTVRPVHRRFEIHSYEEATTTTEVEAALRKVVAEASEGLVLKNPRSPY  487
             +YTLRDRR ALEK V PVHRRFEI  Y EATT  ++E  LR VVAEASEGLVLKNPRSPY
Sbjct  434   RYTLRDRRKALEKVVIPVHRRFEILEYHEATTVADIEKCLRTVVAEASEGLVLKNPRSPY  493

Query  488   RLNERHDDWMKVKPDYMTEFGESLDVVVIGGYYGSGHRGGALSSFLCGLRVD--DSTQAA  545
             RLNERHDDWMKVKP+YMTEFGESLD+VVIGGYYGSGHRGG LSSFLCGLRVD  +++Q A
Sbjct  494   RLNERHDDWMKVKPEYMTEFGESLDLVVIGGYYGSGHRGGNLSSFLCGLRVDEQNASQGA  553

Query  546   E--KCWSFCKVGGGFTAADYQEVRHHTDGKWKAWDAKKPPTTFIELAGGDAQHERPDMWI  603
             +  KCWSFCKVGGGFTAADYQEVRHHTDGKW  WD KKPPT  IELAGGDAQ+ERPDMWI
Sbjct  554   DPMKCWSFCKVGGGFTAADYQEVRHHTDGKWNEWDPKKPPTNLIELAGGDAQYERPDMWI  613

Query  604   KPSDSIVLCVKAASVAISDQFRMGLTLRFPRFKKLRKDKDWKSALSVQEFLDLKSNAEQE  663
             KPSDSIVLCVKAASV++SDQFRMGLTLRFPRFKKLRKDK+WKSALS+QEFLDLKSNAEQE
Sbjct  614   KPSDSIVLCVKAASVSVSDQFRMGLTLRFPRFKKLRKDKNWKSALSIQEFLDLKSNAEQE  673

Query  664   HREKEFSVDNSR-TKRVKRTTKKPLTVAGYDDNIDVQYLGPSGHVFDELNFFIMTESTIP  722
             HREKEFSVDNSR  KR KR  KKPL +AGYD+  + QY GPSGH+FD LNFFIMTEST+P
Sbjct  674   HREKEFSVDNSRKQKRPKRVVKKPLNIAGYDEKENAQYFGPSGHIFDHLNFFIMTESTVP  733

Query  723   EKKTKPQLEQLVKANGGKIYQTRTAAVDTLCIAERRTVKVASLQKSGEQSIIRPSWLIDC  782
             EKKTK +LEQLVKANGGKIYQT TA  DT+CIA+RRTVKVAS+QKSG+ +I+R SWLIDC
Sbjct  734   EKKTKAELEQLVKANGGKIYQTNTAVQDTICIADRRTVKVASVQKSGQTNIVRASWLIDC  793

Query  783   VKQNEIDAGLPDLLLPFEPRHMFFMTEDKEEEVAANVDKFMDSYARDTTVDELKDIFNQM  842
             VKQNE DAGLPDLLLPFEPRHMFF  ED++E++  NVD+FMDSYARDTT+DELK+I +QM
Sbjct  794   VKQNEKDAGLPDLLLPFEPRHMFFTLEDQDEDIKLNVDRFMDSYARDTTIDELKEILDQM  853

Query  843   EQNQKQFDHAPDSETIQRVEARIQEKVNAGYTVPCGWLFRGLKFYFYS----NKDHPDEP  898
             ++ ++Q     D  +I ++E  +Q+K+N+GYT PCGWLFRGL F+F S    N +   +
Sbjct  854   QETEEQTHRPLDPHSIHKIETHVQDKINSGYTAPCGWLFRGLTFFFPSSNPVNGEGSSDS  913

Query  899   TSREPRKEDQRLQFARNTARFAGAESASSFKSSGTTHVIVDPDNLSSADISSLRKSLA  956
              S  P+ ED RL  ARNTA+F GA + +S KSS  THVIV+ + ++SA+ISSLRK++A
Sbjct  914   DSSIPKTEDIRLTLARNTAQFGGASTVTSLKSSSVTHVIVNTEKITSAEISSLRKTVA  971
```

Fig. S4 BLASTp sequence alignment of Pcr2372 of *P. crustosum* (Query) with putative DNA ligase 4 of *Penicillium brasilianum* (OOQ89364.1)

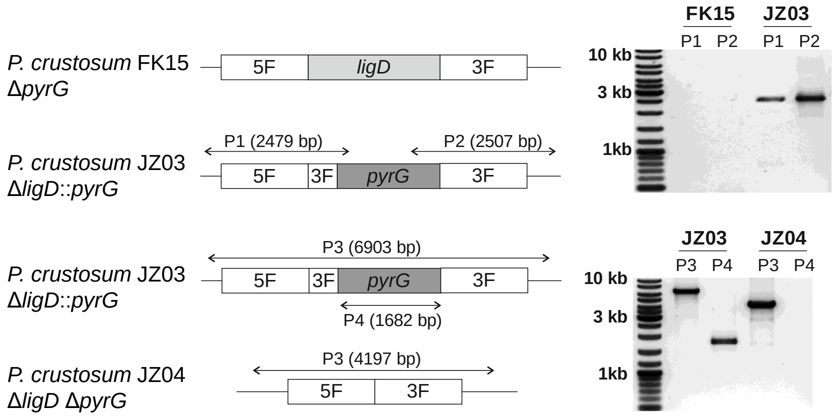

Primers used for amplification of partial fragments P1 – P4:

P1: 5ligDver_f2 and pyrGver_r1
P2: pyrGver_f1 and 3ligDver_r2
P3: JZ05_5pyrG_f and JZ08_3pyrG_r
P4: 5ligDver_f2 and 3ligDver_r2

Fig. S5 Schematic illustration of *ligD* (*pcr2372*) deletion in *P. crustosum* FK15 and PCR verification of JZ03 (Δ*ligD*::*pyrG*) and JZ04 (Δ*ligD*Δ*pyrG*) by amplification of different partial fragments (P1–P4) from genomic DNA. Transformants were verified using primers binding both outside of the deletion construct and in the *pyrG* sequence for P1 and P2, outside the deletion construct for P3, and in the *pyrG* sequence for P4. Primer sequences and their corresponding Primer-IDs are given in Supplemental Table S3. (5F: upstream flanking region, 3F: downstream flanking region)

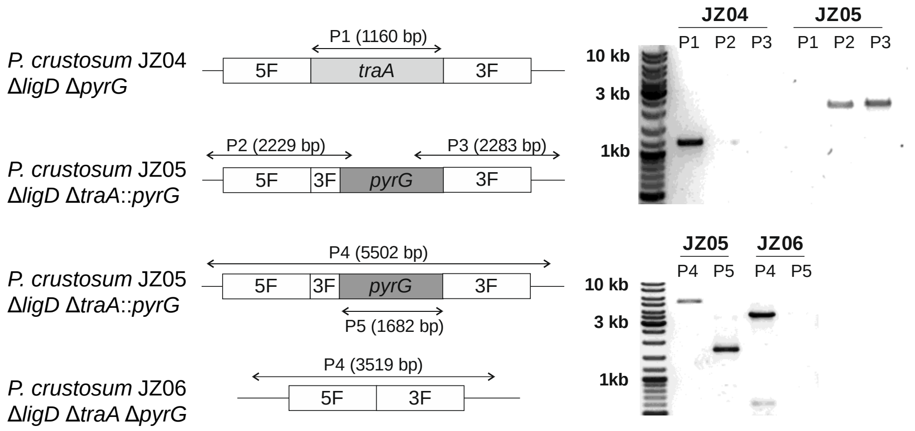

Primers used for amplification of partial fragments P1 – P5:

P1: JZ43_traAscr_f and JZ44_traAscr_r
P2: JZ38_traAdo_v_r2 and JZ37_pyrG_ver2
P3: JZ36_pyrGver1 and JZ63_traAdo_v_r
P4: JZ38_traAdo_v_r2 and JZ63_traAdo_v_r
P5: JZ05_5pyrG_f and JZ08_3pyrG_r

Fig. S6 Schematic illustration of *traA* (*pcr11009*) deletion in *P. crustosum* JZ04 and PCR verification of JZ05 (Δ*traA*::*pyrG*) and JZ06 (Δ*traA*Δ*pyrG*) by amplification of different partial fragments (P1–P5) from genomic DNA. The presence of *traA* was verified with primers binding in the *traA* sequence for P1. Transformants were confirmed using primers binding both outside of the deletion construct and in the *pyrG* sequence for P2 and P3, outside the deletion construct for P4, and in the *pyrG* sequence for P5. Primer sequences and their corresponding Primer-IDs are given in Supplemental Table S3. (5F: upstream flanking region, 3F: downstream flanking region)

Sequence ID: Query_1191009 Length: 119 Number of Matches: 1
Range 1: 1 to 119

Score	Expect	Identities	Gaps	Strand	Frame
220 bits(119)	4e-63()	119/119(100%)	0/119(0%)	Plus/Plus	

```
Query  1    ACATACGACCATAGGGTGTGGAAAACAGGGCTTCCCGTCCGCTCAGCCGTACTTAAGCCA  60
            ||||||||||||||||||||||||||||||||||||||||||||||||||||||||||||
Sbjct  1    ACATACGACCATAGGGTGTGGAAAACAGGGCTTCCCGTCCGCTCAGCCGTACTTAAGCCA  60

Query  61   CACGCCGGTGAGTTAGTAGTTGGGTGGGTGACCACCAGCGAATCCTCACTGTTGTATGT  119
            |||||||||||||||||||||||||||||||||||||||||||||||||||||||||||
Sbjct  61   CACGCCGGTGAGTTAGTAGTTGGGTGGGTGACCACCAGCGAATCCTCACTGTTGTATGT  119
```

Fig. S7 BLASTn sequence alignment of 5S rRNA promoter of *P. crustosum* (Query) with 5S rRNA promoter sequence of *P. chrysogenum* deposited in the 5SRNAdb (Szymanski et al. 2016)

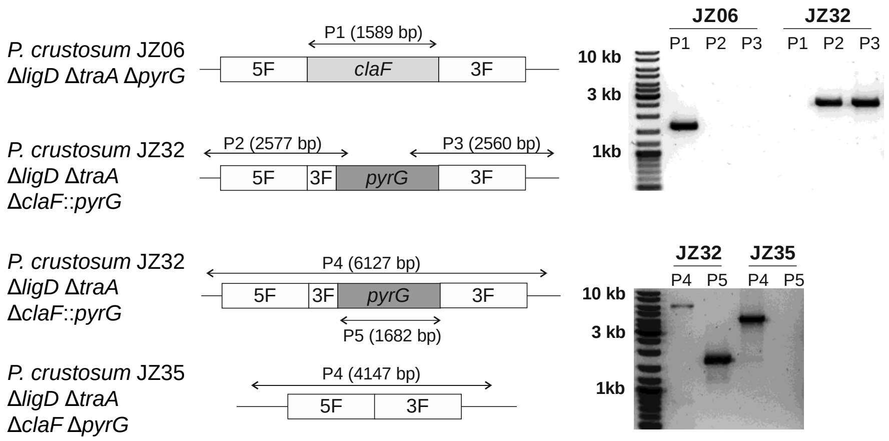

Primers used for amplification of partial fragments P1 – P5:
P1: JZ117_claF_5F_V and JZ100_claF_5F_R2
P2: JZ161_claF_5F_V and JZ37_pyrG_ver2
P3: JZ36_pyrGver1 and JZ118_claF_3F_V
P4: JZ161_claF_5F_V and JZ118_claF_3F_V
P5: JZ05_5pyrG_f and JZ08_3pyrG_r

Fig. S8 Schematic illustration of *claF* (*pcr3094*) deletion in *P. crustosum* JZ06 and PCR verification of JZ32 (Δ*claF*::*pyrG*) and JZ35 (Δ*claF*Δ*pyrG*) by amplification of different partial fragments (P1–P5) from genomic DNA. The presence of *claF* was verified with primers binding in the *claF* sequence for P1. Transformants were confirmed using primers binding both outside of the deletion construct and in the *pyrG* sequence for P2 and P3, outside the deletion construct for P4, and in the *pyrG* sequence for P5. Primer sequences and their corresponding Primer-IDs are given in Supplemental Table S3. (5F: upstream flanking region, 3F: downstream flanking region)

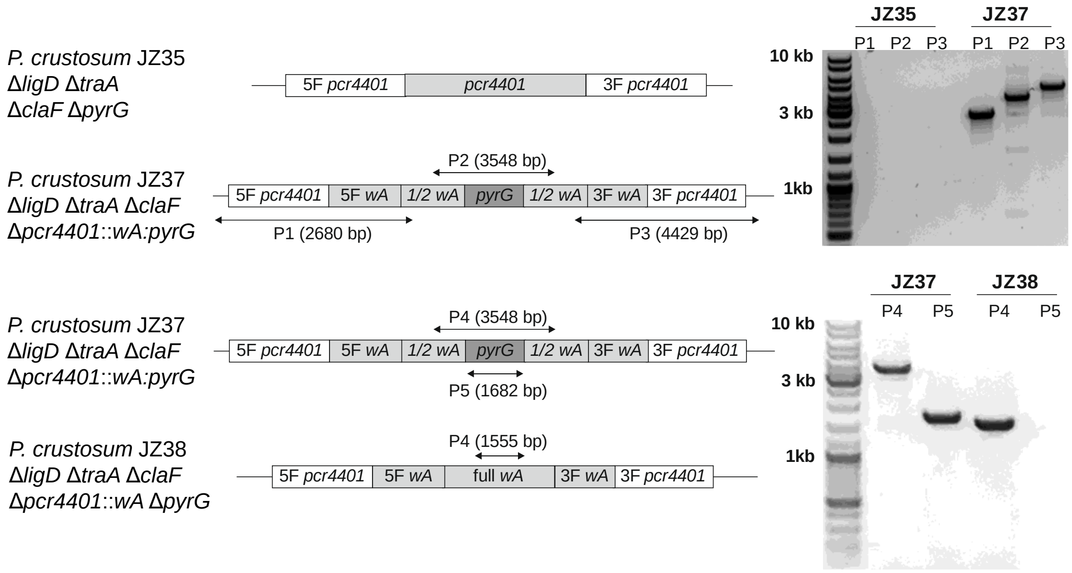

Primers used for amplification of partial fragments P1 – P5:

P1: JZ177_4401ver_f and JZ128_wA1_r
P2: JZ75_wAdown_f and JZ123_wA_Fra2_r
P3: JZ133_wA4_f and JZ178_4401ver_r
P4: JZ75_wAdown_f and JZ123_wA_Fra2_r
P5: JZ05_5pyrG_f and JZ08_3pyrG_r

Fig. S9 Schematic illustration of *wA* expression in *P. crustosum* JZ35 and PCR verification of JZ37 (*wA*::*pyrG*) and JZ38 (*wA*Δ*pyrG*) by amplification of different partial fragments (P1–P5) from genomic DNA. Transformants were confirmed by amplification of the upstream and downstream sequence with primers binding outside of the deletion cassette and in the *wA* gene for P1 and P3, and by amplification of parts of the *wA* gene with or without the *pyrG* sequence for P2 and P4. The presence of *pyrG* was verified with primers binding in the *pyrG* sequence for P5. Primer sequences and their corresponding Primer-IDs are given in Supplemental Table S3. (5F: upstream flanking region, 3F: downstream flanking region)

Sequence ID: Query_67415 Length: 360 Number of Matches: 1
Range 1: 10 to 353

Score	Expect	Method	Identities	Positives	Gaps	Frame
491 bits(1264)	7e-178()	Compositional matrix adjust.	251/361(70%)	283/361(78%)	25/361(6%)	+1

```
Query  307   PEVGAMGSTPGDLTPGVPASLLSPSFTPPATPGGTLNTE----RLLQQISPQPVSH----  462
             P++   G   G+ TP +P SL+SP+FTPPATPGGTLN       +L Q      + H
Sbjct  10    PKLPPRGIATGEQTPALPESLISPAFTPPATPGGTLNLNLSPTAVLHQTQAADIDHKSKA  69

Query  463   AKPPKLLPRLPNVECIVRARIPTTTGAEMFLHLYHNDIDNKEHLAIVFGNTIRSRSLDRV  642
             AK PKLLP+LP VECIVRARIPTT GAEMFLHLYHND+D KEHLAIVFGN IRSRSLD V
Sbjct  70    AKGPKLLPQLPAVECIVRARIPTTNGAEMFLHLYHNDLDGKEHLAIVFGNNIRSRSLDSV  129

Query  643   KPGETEMDRMIRGAYIGKLHPGRVSSWHDSTQGSATDRSIEGSEGGAVHNTESMQERLNE  822
             +PGE+EMDRMIRGAY+GKLHPGRVSS +D                G+V    + +     E
Sbjct  130   RPGESEMDRMIRGAYVGKLHPGRVSSRYDDELA------------GSVSTPKQI-----E  172

Query  823   APLVRIHSECYTGETAWSARCDCGEQLDEAARLMSLPMETLNEIASQQSRSVPSNVSGGV  1002
              PLVRIHSECYTGETAWSARCDCGEQLDEAARLMS P+E L   A  + +S+ S+ +GGV
Sbjct  173   PPLVRIHSECYTGETAWSARCDCGEQLDEAARLMSFPVEDLASDAPPEVQSLSSHSTGGV  232

Query  1003  IIYLRQEGRGIGLGEKLKAYNLQDLGSDTVEANLLLRHPADARSYGLATAMLVDLGLGKD  1182
             I+YLRQEGRGIGLGEKLKAYNLQDLGSDTVEANLLLRHPADARSYGLATA+L DLG G D
Sbjct  233   IVYLRQEGRGIGLGEKLKAYNLQDLGSDTVEANLLLRHPADARSYGLATAILEDLGCGVD  292

Query  1183  ANPHGIRLLTNNPDKVRAIEGPGREVIVKDRVPMVPLAWQTGGKMGIKSSEVEGYLRTKA  1362
             A P GIRLLTNNPDKVRAIEGP REV+VK+RVPM+PLAW+TGG+ GIKSSE+EGYL+TK
Sbjct  293   AIPEGIRLLTNNPDKVRAIEGPNREVLVKERVPMIPLAWRTGGQKGIKSSEIEGYLQTKI  352

Query  1363  S  1365
             S
Sbjct  353   S  353
```

Fig. S10 BLASTx sequence alignment of the gene product of *riboB* from *A. nidulans* (AN0670.2, Query) with Pcr11223 from *P. crustosum*

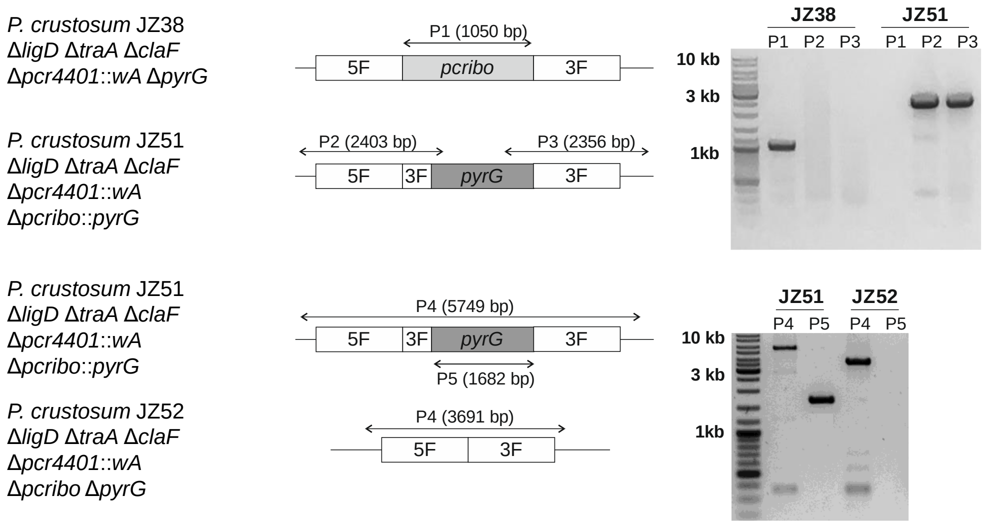

Primers used for amplification of partial fragments P1 – P5:

P1: JZ269_riboGen_f and JZ270_riboGen_r
P2: JZ267_ribo5V_f and JZ37_pyrG_ver2
P3: JZ36_pyrGver1 and JZ268_ribo3V_r
P4: JZ267_ribo5V_f and JZ268_ribo3V_r
P5: JZ05_5pyrG_f and JZ08_3pyrG_r

Fig. S11 Schematic illustration of *pcribo* (*pcr11223*) deletion in *P. crustosum* JZ38 and PCR verification of JZ51 (Δ*pcribo*::*pyrG*) and JZ52 (Δ*pcribo*Δ*pyrG*) by amplification of different partial fragments (P1–P5) from genomic DNA. The presence of *pcribo* was verified with primers binding in the *pcribo* sequence for P1. Transformants were confirmed using primers binding both outside of the deletion construct and in the *pyrG* sequence for P2 and P3, outside the deletion construct for P4, and in the *pyrG* sequence for P5. Primer sequences and their corresponding Primer-IDs are given in Supplemental Table S3. (5F: upstream flanking region, 3F: downstream flanking region)

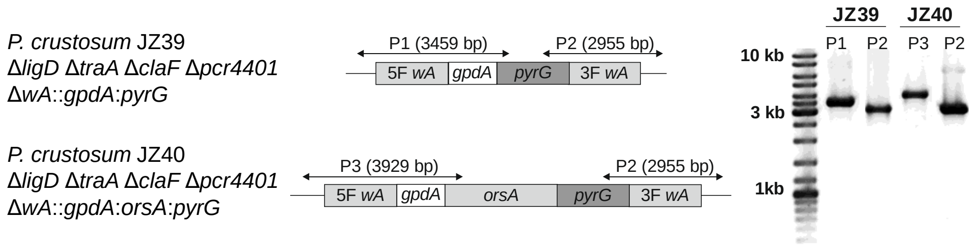

Primers used for amplification of partial fragments P1 – P3:

P1: JZ177_4401ver_f and JZ37_pyrG_ver2
P2: JZ36_pyrGver1 and JZ178_4401ver_r
P3: JZ177_4401ver_f and JZ243_pPX26ver_r

Fig. S12 Schematic illustration of *orsA* expression in *P. crustosum* JZ38 and PCR verification of JZ39 (*gpdA*(p)::*pyrG*, empty vector control) and JZ40 (*gpdA*(*p*)-*orsA*::*pyrG*) by amplification of different partial fragments (P1–P2) from genomic DNA. Transformants were verified using primers binding outside of the deletion cassette and in the *pyrG* sequence for P1 and P2, or in the *orsA* gene for P3, respectively. Primer sequences and their corresponding Primer-IDs are given in Supplemental Table S3. (5F: upstream flanking region, 3F: downstream flanking region)

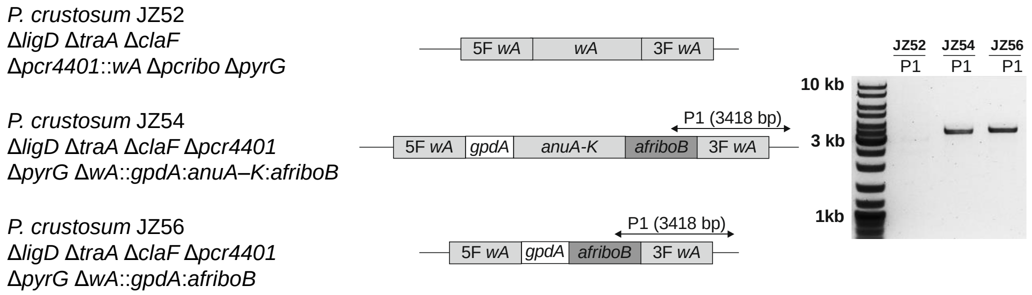

Primers used for amplification of partial fragment P1:
P1: JZ57_An_traB_5Vr and JZ59_An_traB_GVr

Fig. S13 Schematic illustration of annullatin (*anu*) cluster expression in *P. crustosum* JZ52 and PCR verification of JZ54 (*gpdA*(*p*)-*anuA–K*::*afriboB*) and JZ56 (*gpdA*(*p*)::*afriboB*, empty vector control) by amplification of downstream partial fragment (P1) from genomic DNA. Transformants were verified using primers binding outside of the deletion cassette and in the *afriboB* sequence for P1. Primer sequences and their corresponding Primer-IDs are given in Supplemental Table S3. (5F: upstream flanking region, 3F: downstream flanking region)

References

Chiang YM, Ahuja M, Oakley CE, Entwistle R, Asokan A, Zutz C, Wang CC, Oakley BR (2016) Development of genetic dereplication strains in *Aspergillus nidulans* results in the discovery of aspercryptin. Angew Chem Int Ed 55:1662–1665. doi: 10.1002/anie.201507097

Kindinger F, Nies J, Becker A, Zhu T, Li S-M (2019) Genomic locus of a *Penicillium crustosum* pigment as an integration site for secondary metabolite gene expression. ACS Chem Biol 14:1227–1234. doi: 10.1021/acschembio.9b00164

Liang Y, Han Y, Wang C, Jiang C, Xu J-R (2018) Targeted deletion of the *USTA* and *UvSLT2* genes efficiently in *Ustilaginoidea virens w*ith the CRISPR-Cas9 system. Front Plant Sci 9:699. doi: 10.3389/fpls.2018.00699

Szymanski M, Zielezinski A, Barciszewski J, Erdmann VA, Karlowski WM (2016) 5SRNAdb: an information resource for 5S ribosomal RNAs. Nucleic Acids Res 44:D180-3. doi: 10.1093/nar/gkv1081

Xiang P, Kemmerich B, Yang L, Li S-M (2022) Biosynthesis of annullatin D in *Penicillium roqueforti* implies oxidative lactonization between two hydroxyl groups catalyzed by a BBE-like enzyme. Org Lett 24:6072–6077. doi: 10.1021/acs.orglett.2c02438

Xiang P, Li S-M (2022) Formation of 3-orsellinoxypropanoic acid in *Penicillum crustosum* is catalyzed by a bifunctional nonreducing polyketide synthase. Org Lett 24:462–466. doi: 10.1021/acs.orglett.1c04189

Yin WB, Chooi YH, Smith AR, Cacho RA, Hu Y, White TC, Tang Y (2013) Discovery of cryptic polyketide metabolites from dermatophytes using heterologous expression in *Aspergillus nidulans*. ACS Synth Biol 2:629–634. doi: 10.1021/sb400048b
